# Elevated protein synthesis in microglia causes autism-like synaptic and behavioral aberrations

**DOI:** 10.1038/s41467-020-15530-3

**Published:** 2020-04-14

**Authors:** Zhi-Xiang Xu, Gyu Hyun Kim, Ji-Wei Tan, Anna E. Riso, Ye Sun, Ethan Y. Xu, Guey-Ying Liao, Haifei Xu, Sang-Hoon Lee, Na-Young Do, Chan Hee Lee, Amy E. Clipperton-Allen, Soonwook Kwon, Damon T. Page, Kea Joo Lee, Baoji Xu

**Affiliations:** 1 Department of Neuroscience, The Scripps Research Institute Florida, Jupiter, FL 33458 USA; 2 Synaptic Circuit Plasticity Lab, Korea Brain Research Institute, Daegu, 41062 Korea; 3 The Harriet L. Wilkes Honors College, Florida Atlantic University, Jupiter, FL 33458 USA; 4 Integrative Program in Biology and Neuroscience, Florida Atlantic University, Jupiter, FL 33458 USA; 5 Advanced Neural Imaging Center, Department of Structure & Function of Neural Network, Korea Brain Research Institute, Daegu, 41062 Korea; 6 Department of Anatomy, Catholic University of Daegu, Daegu, 42472 Korea

**Keywords:** Cellular neuroscience, Autism spectrum disorders, Molecular neuroscience

## Abstract

Mutations that inactivate negative translation regulators cause autism spectrum disorders (ASD), which predominantly affect males and exhibit social interaction and communication deficits and repetitive behaviors. However, the cells that cause ASD through elevated protein synthesis resulting from these mutations remain unknown. Here we employ conditional overexpression of translation initiation factor eIF4E to increase protein synthesis in specific brain cells. We show that exaggerated translation in microglia, but not neurons or astrocytes, leads to autism-like behaviors in male mice. Although microglial eIF4E overexpression elevates translation in both sexes, it only increases microglial density and size in males, accompanied by microglial shift from homeostatic to a functional state with enhanced phagocytic capacity but reduced motility and synapse engulfment. Consequently, cortical neurons in the mice have higher synapse density, neuroligins, and excitation-to-inhibition ratio compared to control mice. We propose that functional perturbation of male microglia is an important cause for sex-biased ASD.

## Abstract (web summary)

The main cell types involved in autism spectrum disorders through elevated protein synthesis are not well identified. Here, the authors show that overexpression of translation initiation factor eIF4E in microglia results in autism-like behaviour in male, but not female, mice.

## Introduction

Autism spectrum disorders (ASD) are a group of neurodevelopmental disorders with deficits in two core domains: social interaction and communication, and repetitive or restrictive behaviors^1,2^. ASD are frequently associated with comorbidities, such as intellectual disability and anxiety^3,4^. Prevalence of ASD is 1 in 59 children under 8 years of age, and males are four times more likely than females to be identified with ASD^5^. The biological basis for this sex bias is not clear. There is a strong genetic basis for ASD^6–8^. One recognized common biochemical pathway underlying ASD is dysregulation of protein synthesis^3^.

Eukaryotic mRNAs contain a modified guanosine (m^7^Gppp), termed a cap, at their 5′ end. Translation of mRNAs requires binding of a translation initiation factor eIF4E to the cap. This factor interacts with the scaffolding protein eIF4G, which is essential for formation of the translation initiation complex by bridging an mRNA to the ribosome^9^. Hypophosphorylated forms of eIF4E-binding proteins (4E-BPs) repress translation initiation by sequestering eIF4E and consequently disrupting formation of the translation initiation complex^9,10^. Although most mRNAs need basal amounts of eIF4E to be translated, eIF4E preferentially increases translation of a selective set of mRNAs with extensive secondary structures at their 5′ untranslated regions^11^. The mammalian target of rapamycin complex 1 (mTORC1) phosphorylates 4E-BPs and releases eIF4E from the 4E-BP complex, thus stimulating translation^9,10^.

There are four main negative regulators upstream of mTORC1: PTEN (phosphatase and tensin homolog), TSC1 (tuberous sclerosis complex 1), TSC2 and neurofibromatosis type 1^3^. Inactivating mutations in the genes for these proteins cause ASD in a subset of patients^12,13^. Furthermore, fragile X syndrome is caused by silencing of the *FMR1* gene, which encodes fragile X mental retardation protein (FMRP)^14^, and the mTORC1-eIF4E pathway is over-activated in fragile X syndrome patients diagnosed with ASD^15^. These single-gene disorders account for over 3% of all ASD cases^3^. These discoveries suggest that elevated translation might cause ASD in a subset of individuals. Importantly, a causal relationship between elevated translation and ASD-like behaviors has been recently established in mice. Deletion of the 4E-BP2-coding *Eif4ebp2* gene or overexpression of eIF4E under the promoter of beta tubulin (βT-Eif4e) increases protein synthesis in the mouse brain and leads to ASD-like behaviors^16,17^.

Microglia are derived from myeloid progenitors generated in the yolk sac and migrate into the brain when neurons and other glial cells only begin to be produced during early embryogenesis^18,19^. After the closure of the blood brain barrier, microglia self-renew without the contribution of circulating monocytes in the healthy brain^20,21^. Until recently, the primary function of microglia was thought to migrate to inflammation sites and engulf debris from dead or dying cells^22^. Recent work indicates that homeostatic microglia also play important roles in synaptic development and function^23–26^.

As synthesis of synaptic proteins is necessary for enduring synaptic plasticity and synaptic dysfunction can lead to ASD, it has been proposed that inactivating mutations in negative translation regulators cause ASD by enhancing translation of mRNAs in neurons^27,28^. Because mRNA translation is elevated in all cells of the body in *Eif4ebp2* knockout and transgenic βT-Eif4e mice^16,17^, it remains, however, to be determined whether translational dysregulation in neurons is sufficient to cause ASD and whether translational dysregulation in glial cells contributes to autism manifestations. In this study, we elevated mRNA translation by overexpressing eIF4E and showed that exaggerated translation in microglia is sufficient to cause ASD-like phenotypes in mice via its detrimental impact on microglia-neuron interactions.

## Results

### Intact sociability in mice with elevated neuronal translation

**Fig. 1:**
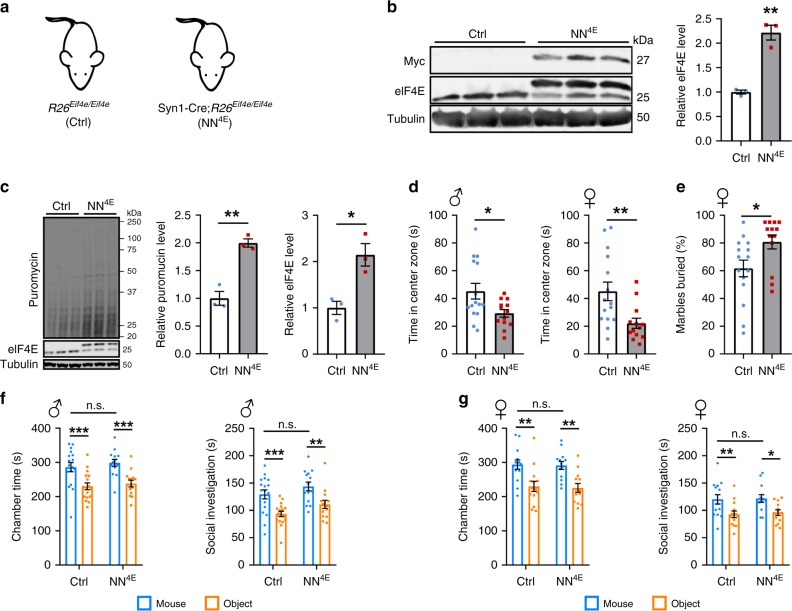
Neuronal eIF4E overexpression elevates anxiety without altering social interaction. **a** Syn1-Cre;*R26*^*Eif4e/Eif4e*^ (NN^4E^) mice overexpress eIF4E in neurons, while *R26*^*Eif4e/Eif4e*^ mice serve as controls (Ctrl). **b** Levels of eIF4E in hippocampal extracts prepared from control and NN^4E^ mice. The eIF4E immunoblot reveals both endogenous eIF4E (lower band) and overexpressed eIF4E-Myc (upper band). Alpha tubulin was used as a loading control. *n* = 3 per genotype. **p = 0.0015 by two-sided *t*-test. **c** Protein synthesis in cultured hippocampal neurons as revealed by puromycin incorporation. *n* = 3 mice per genotype. **p = 0.0023 and *p = 0.015 by two-sided *t*-test. **d** Time spent in the central zone during the first 5 min in open field tests. Male: 15 control mice and 13 NN^4E^ mice; Female: 15 control mice and 13 NN^4E^ mice. **p = 0.0076, *p = 0.0218 by two-sided *t*-test. **e** Percentage of marbles buried. n = 15 control mice and 13 NN^4E^ mice. *p = 0.0247 by two-sided *t*-test. **f**, **g** Sociability of male (**f**) and female (**g**) NN^4E^ mice as revealed in three-chamber sociability tests. Male: 17 control mice and 14 NN^4E^ mice; Female: 14 control mice and 13 NN^4E^ mice. Two-way analysis of variance (ANOVA) with Fisher’s LSD post-hoc test: *p < 0.05, **p < 0.01, ***p < 0.001, and n.s. not significant. All data are shown as mean ± s.e.m. Source data are provided as a Source Data file.

We generated a conditional eIF4E overexpression allele at the *Rosa26* locus (*R26*^*Eif4e*^) that expresses Myc-tagged eIF4E in a Cre-dependent manner (Supplementary Fig. 1a, b). eIF4E-Myc interacted with eIF4G as efficiently as untagged eIF4E (Supplementary Fig. 1c), indicating that the Myc tag does not interfere with the function of eIF4E. As it is generally believed that elevated protein synthesis in neurons causes ASD-like behaviors^27^, we first overexpressed eIF4E in neurons by crossing *R26*^*Eif4e*^ mice with Syn1-Cre mice, which selectively express Cre in neurons as early as embryonic day 12.5^29^, to generate Syn1-Cre;*R26*^*Eif4e/Eif4e*^ mice (termed NN^4E^ mice thereafter) (Fig. 1a). Levels of total eIF4E (eIF4E + eIF4E-Myc) in the NN^4E^ hippocampus were more than twice as high as those in control mice (Fig. 1b). Interestingly, neuronal transgenic eIF4E expression significantly reduced endogenous eIF4E levels (Fig. 1b and Supplementary Fig. 2a). In fact, we found that one copy of the *R26*^*Eif4e*^ allele was not sufficient to significantly increase levels of total eIF4E in the brain due to this negative feedback regulation (1.01 ± 0.10 for Syn1-Cre;*R26*^*Eif4e/+*^ vs. 1.00 ± 0.09 for *R26*^*Eif4e/+*^, *p* = 0.92, *n* = 5 mice per genotype). Using surface sensing of translation (SUnSET) which measures incorporation of puromycin into nascent polypeptides^30^, we found that protein synthesis rate was doubled in cultured NN^4E^ hippocampal neurons over control neurons (Fig. 1c). These results indicate that neuronal protein synthesis is increased in NN^4E^ mice.

We assessed if elevated protein synthesis in neurons leads to abnormal behaviors in mice. As revealed in rotarod and open-field tests (Supplementary Fig. 2b, c), NN^4E^ mice had intact motor function. We examined anxiety-like behaviors by performing open field, elevated plus maze, and light-dark box tests. Anxious mice tend to avoid open, exposed, and brightly illuminated areas. Although NN^4E^ and control mice spent comparable time in the light chamber in light-dark box tests (Supplementary Fig. 2e), NN^4E^ mice of both sexes spent significantly less time in the center of the open field than control mice in the first 5 min of a 30-min open field test (Fig. 1d). In addition, male NN^4E^ mice stayed longer in the closed arm of an elevated plus maze than control mice (Supplementary Fig. 2d). These results indicate that elevated neuronal protein synthesis increases anxiety. In agreement with the requirement of protein synthesis for long-term memory^31^, NN^4E^ mice showed more freezing in the contextual chamber 24 h after fear condition training than control mice (Supplementary Fig. 2g). Conversely, working memory was unaffected in NN^4E^ mice as measured by T-maze alternation (Supplementary Fig. 2f). We then ran marble burying and 3-chamber sociability tests to determine if elevated neuronal protein synthesis leads to repetitive behaviors and deficits in social interaction. Although female NN^4E^ mice showed increased repetitive behaviors in marble burying tests relative to control mice (Fig. 1e), male NN^4E^ mice were unaffected (Supplementary Fig. 2h). Unexpectedly, NN^4E^ mice of both sexes stayed longer in the chamber with a holder holding a stranger mouse than in the chamber with an inanimate object (empty holder) and spent more time in investigating the stranger mouse than the empty holder, as did control mice in 3-chamber sociability tests (Fig. 1f, g). These behavioral results indicate that elevated neuronal protein synthesis does not lead to deficits in social interaction associated with ASD. This suggests that social interaction impairments observed in *Eif4ebp2* knockout and transgenic βT-Eif4e mice^16,17^ are attributable to elevated protein synthesis in glial cells.

### Elevated microglial translation leads to ASD-like behavior

We next overexpressed eIF4E in astrocytes (Supplementary Fig. 3a–c), which is the largest population of glia in the brain, using a Cre transgene driven by the astrocyte-specific promoter for glial fibrillary acidic protein (GFAP-Cre)^32^ which becomes active during late embryogenesis and the first postnatal week^33^. We found that astrocytic eIF4E overexpression did not alter repetitive and social behaviors in mice of either sex (Supplementary Fig. 3d–f). Thus, we shifted our efforts to microglia, which make up ~10% of brain cells^34^.

We employed *Cx3cr1*^*CreER*^ mice^25^ to overexpress eIF4E in microglia. To determine the efficiency for tamoxifen to activate Cre recombinase in microglia, we crossed *Cx3cr1*^*CreER/+*^ mice to *Rosa26*^*Ai9/+*^ mice^35^ to generate *Cx3cr1*^*CreER/+*^;*Rosa26*^*Ai9/+*^ mice. A single tamoxifen injection (180 mg/kg, subcutaneously) was administered to *Cx3cr1*^*CreER/+*^;*Rosa26*^*Ai9/+*^ mice at P0. Tamoxifen activated CreER to excise the loxP-flanked transcription blocker of the *Rosa26*^*Ai9*^ allele in microglia, leading to tdTomato expression (Supplementary Fig. 4a). There is nearly 100% colocalization (*n* = 3 mice) between tdTomato and the microglial marker, ionized calcium binding adapter molecule 1 (Iba1), in the hippocampus and cortex (Supplementary Fig. 4b), indicating extremely high specificity in the expression of the *Cx3cr1*^*CreER*^ gene. Furthermore, the recombination activity of the CreER protein is tightly controlled by tamoxifen, as we did not detect tdTomato expression in *Cx3cr1*^*CreER/+*^;*Rosa26*^*Ai9/+*^ mice in the absence of tamoxifen injection (Supplementary Fig. 4c). This observation indicates that a single tamoxifen injection in newborn pups is sufficient to activate Cre recombinase selectively in microglia of *Cx3cr1*^*CreER*^ mice.

**Fig. 2:**
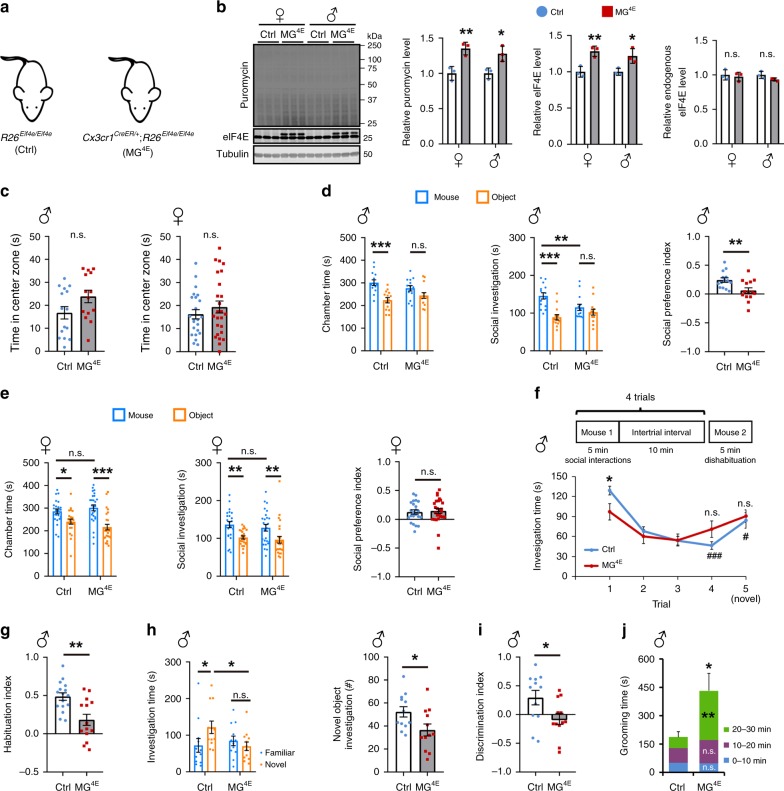
Overexpression of eIF4E in microglia leads to social deficits. **a** Genotypes of control (Ctrl) and MG^4E^ mice. **b** Microglial eIF4E overexpression and protein synthesis in cultured microglia isolated from both sexes of control and MG^4E^ mice. *n* = 3 per condition. Two-sided *t*-test: puromycin, female **p = 0.0099 and male *p = 0.0217; eIF4E, female **p = 0.0088 and male *p = 0.0293. **c** Time spent in the central zone during the first 5 min of open field tests. Male: 15 control mice and 14 MG^4E^ mice; Female: 23 control mice and 25 MG^4E^ mice. n.s. not significant by two-sided *t*-test. **d**, **e** Sociability of MG^4E^ mice. Male: 14 control mice and 14 MG^4E^ mice; Female: 23 control mice and 25 MG^4E^ mice. Two-way ANOVA with Fisher’s LSD post-hoc test for chamber time and social investigation: *p < 0.05; **p < 0.01; ***p < 0.001; n.s. not significant. Two-sided *t*-test for social preference index: male, **p = 0.0067; female, *p* = 0.7440. **f** Social habituation test. *n* = 16 male control and 14 male MG^4E^ mice. Two-way ANOVA with Fisher’s LSD post-hoc test: *p = 0.0159 between control and MG^4E^ mice in trial 1; ^###^*p* < 0.001 between trial1 and trial4 in control mice; ^#^*p* = 0.0334 between trial 4 and trial 5 in control mice; n.s. not significant for comparisons of trial 1 vs. trial 4 and trial 4 vs. novel in MG^4E^ mice. **g** Habituation index. **p = 0.0013 by two-sided *t*-test. **h**, **i** Novel object recognition. *n* = 12 control mice and 12 MG^4E^ mice. Two-way ANOVA with Fisher’s LSD post-hoc test: *p < 0.05 and n.s. not significant (**h**). Two-sided *t*-test: *p = 0.0297 (**i**). **j**, Self-grooming. The 30-min self-grooming test was divided into three 10-min segments. *n* = 6 mice per genotype. Two-way ANOVA with Fisher’s LSD post-hoc test was used for each 10-min segment, whereas two-sided *t*-test was used for the whole 30-min test period. *p = 0.0276; **p = 0.0016; n.s. not significant. Source data are provided as a Source Data file.

Newborn pups from *R26*^*Eif4e/Eif4e*^ × *Cx3cr1*^*CreER/+*^;*R26*^*Eif4e/Eif4e*^ crosses were treated with tamoxifen (180 mg/kg, subcutaneously) to generate control (*R26*^*Eif4e/Eif4e*^) and MG^4E^ (*Cx3cr1*^*CreER/+*^;*R26*^*Eif4e/Eif4e*^) mice that should express eIF4E-Myc in microglia (Fig. 2a). Immunoblotting analysis of brain lysates revealed eIF4E-Myc expression in MG^4E^ mice (Supplementary Fig. 4d). Immunohistochemistry revealed that eIF4E levels were lower in microglia than in neurons in control mice, but the transgenic overexpression increased microglial eIF4E levels more than two folds (Supplementary Fig. 5a). To further assess microglial eIF4E overexpression, we employed an immunopanning method^36^ to purify microglia from control and MG^4E^ mice at P10 (Supplementary Fig. 6). Analysis of purified microglia revealed that two copies of Cre-excised *R26*^*Eif4e*^ alleles increased levels of total eIF4E by 21–28% and protein synthesis rate by 28–35% in both male and female MG^4E^ microglia compared to control microglia (Fig. 2b). Interestingly, eIF4E-Myc expression did not reduce endogenous eIF4E levels in microglia (Fig. 2b), as it did in neurons (Supplementary Fig. 2a) and astrocytes (Supplementary Fig. 3c). These results indicate that transgenic eIF4E-Myc expression elevates microglial protein synthesis to a similar extent in both sexes of mice.

There is a difference in the extent of microglial eIF4E overexpression between the two aforementioned assays. To understand the discrepancy, we established cultures enriched for neurons, astrocytes, or microglia. To our surprise, eIF4E levels in cultured microglia were higher than those in cultured neurons (Supplementary Fig. 5b), although eIF4E levels are higher in neurons than in microglia in vivo on the basis of immunohistochemistry (Supplementary Fig. 5a). This result indicates that cell culturing somehow upregulates microglial eIF4E expression, which should cause underestimation of transgenic eIF4E expression. Therefore, it is likely that microglia in MG^4E^ mice have more than twice as much eIF4E and protein synthesis as those in control mice, as do neurons in NN^4E^ mice and astrocytes in AC^4E^ mice.

Microglia are the most abundant CNS macrophages, which also include perivascular macrophages, meningeal macrophages and choroid-plexus macrophages^37^. In addition to CNS macrophages, CX3CR1 is expressed in peripheral monocytes and inflammatory macrophages^25,37^. In contrast to fast turnover of peripheral CX3CR1-expressing cells, CNS macrophages are long-living^37^. We ran behavioral tests when MG^4E^ mice were at 2–3 months of age when the peripheral monocytes present at the time of tamoxifen injection should have been turned over, so that peripheral cells express little or no elF4E-Myc in tested mice (Supplementary Fig. 5c).

MG^4E^ mice had normal locomotion in open field tests, indicating normal motor function (Supplementary Fig. 7a). They did not show anxiety-like behaviors in open field tests (Fig. 2c), elevated plus maze tests, or light-dark box tests (Supplementary Fig. 7b, c). Interestingly, MG^4E^ mice displayed sexual dimorphism in 3-chamber sociability tests. Male MG^4E^ mice displayed social interaction deficits, as they spent similar amounts of time investigating a stranger mouse and an inanimate object (Fig. 2d), whereas female MG^4E^ mice had normal social interaction behaviors (Fig. 2e). To further characterize social behaviors of male MG^4E^ mice, we performed social recognition tests in the home cage-like environment, an assay which relies on the mouse’s innate tendency to investigate a novel social partner and decrease the investigation of a known social stimulus (social habituation)^38^. Consistent with the social interaction deficit observed in 3-chamber sociability tests, male MG^4E^ mice spent less time investigating a novel stimulus mouse in trial 1 (Fig. 2f). Furthermore, social habituation (trial 4 vs. trial 1) and dishabituation (novel stimulation mouse vs. trial 4) were impaired in male MG^4E^ mice (Fig. 2f, g), indicating that their ability to adapt to novel social stimuli was attenuated, a phenomenon which has been linked to the pathognomonic social impairment and behavioral rigidity in ASD patients^39^. Again, female MG^4E^ mice performed normally in social recognition tests (Supplementary Fig. 7f, g). These results indicate that elevated protein synthesis in microglia leads to social interaction deficits only in male mice.

We evaluated learning and memory in MG^4E^ mice by conducting contextual fear conditioning and novel object recognition tests. Both sexes of control and MG^4E^ mice showed similar long-term hippocampus-dependent contextual fear memory (Supplementary Fig. 7d). However, male, but not female, MG^4E^ mice spent less time investigating a novel object than control littermates (Fig. 2h, i and Supplementary Fig. 7h), indicating recognition memory deficits in male MG^4E^ mice. Thus, male MG^4E^ mice exhibit selective deficits in cognitive functions.

To determine whether MG^4E^ mice display repetitive behaviors, one of the core domains of ASD, we performed marble-burying and self-grooming tests. Although both sexes of MG^4E^ mice performed normally in marble-burying tests (Supplementary Fig. 7e), male, but not female, MG^4E^ mice spent significantly more time grooming than control mice (Fig. 2j and Supplementary Fig. 7i).

One copy of the *Cx3cr1* gene is disrupted in *Cx3cr1*^*CreER/+*^ mice^25^. To rule out the possibility that the observed ASD-like phenotype in MG^4E^ mice results from *Cx3cr1* haploinsufficiency, we examined the behaviors of WT and *Cx3cr1*^*CreER/+*^ mice in three-chamber sociability tests (Supplementary Fig. 8a–d) and novel object recognition tests (Supplementary Fig. 8e–j). Both male and female *Cx3cr1*^*CreER/+*^ mice were normal in these behavioral tests.

Collectively, these results indicate that elevated protein synthesis in microglia leads to ASD-like phenotypes in mice, including male bias, deficits in social interaction, increased repetitive behaviors, and impaired cognitive functions.

### Elevated protein synthesis alters microglial morphology

**Fig. 3:**
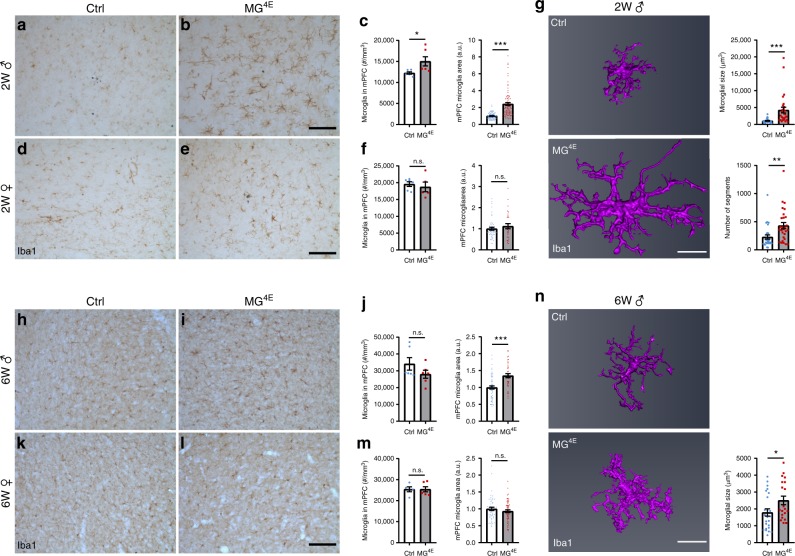
Iba1 immunohistochemistry reveals altered microglial density and morphology in male MG^4E^ mice. **a**–**c** Increased microglial density and size in the mPFC of male MG^4E^ mice. *n* = 7 control mice and 6 MG^4E^ mice. Ten microglia from each mouse were randomly selected for measurement of cell size (cross section area). *p = 0.0342 and ***p < 0.001 by two-sided *t*-test. Scale bar, 100 μm. **d**–**f** Comparable microglial density and size in the mPFC between female control and MG^4E^ mice. *n* = 6 control mice and 5 MG^4E^ mice. Five to ten microglia from each mouse were randomly selected for measurement of cell size (cross section area). n.s. not significant by two-sided *t*-test. Scale bar, 100 μm. **g** Three-dimension reconstruction of microglia in the mPFC of 2-week-old male mice. *n* = 7 mice per group. Three to five microglia from each mouse were reconstructed for determination of cell size (volume) and number of processes. **p = 0.0037 and ***p = 0.0005 by two-sided *t*-test. Scale bar, 20 μm. **h**–**m** Microglia density and size in the mPFC of 6-week-old male (**h**–**j**) and female (**k**–**m**) MG^4E^ mice. Ten microglia in each brain region of each mouse were randomly selected for measurement of cell size (cross section area). Male, *n* = 6 mice per genotype; female, *n* = 5 control mice and 6 MG^4E^ mice. Two-sided *t*-test: ***p < 0.001 and n.s. not significant. Scale bar, 100 µm. **n** Three-dimension reconstruction of microglia in the mPFC of 6-week-old male mice. Four microglia from each mouse were reconstructed. Six control mice and five MG^4E^ mice. *p = 0.0385 by two-sided *t*-test. Scale bar, 20 µm. All data are shown as mean ± s.e.m. Source data are provided as a Source Data file.

We next sought to investigate why microglial eIF4E overexpression increased protein synthesis in both sexes, but only caused ASD-like behaviors in males. We performed Iba1 immunohistochemistry to examine the impact of elevated protein synthesis on microglia in the medial prefrontal cortex (mPFC), hippocampus and striatum, the three brain regions that have been implicated in ASD pathophysiology^40–42^. We found that microglia in the mPFC were more numerous and larger in 2-week-old male MG^4E^ mice than male control littermates (Fig. 3a–c). The same phenotype was observed in the hippocampus and striatum (Supplementary Fig. 9a–f). In contrast, elevated microglial protein synthesis did not affect the density and size of microglia in the mPFC, hippocampus and striatum of 2-week-old female MG^4E^ mice (Fig. 3d–f and Supplementary Fig. 9g–l). Semi-automatic quantitative morphometric 3D measurements of microglia confirmed the increase in size and complexity of microglia in 2-week-old male MG^4E^ mice (Fig. 3g). By 6 weeks of age, microglia in the mPFC, hippocampus and striatum in male MG^4E^ mice remained larger than those of male control mice; however, their density became normal or slightly lower compared to control mice (Fig. 3h–j, n and Supplementary Fig. 10a–f). Again, elevated protein synthesis had no detectable effect on microglial size and density in 6-week-old female mice (Fig. 3k–m and Supplementary Fig. 10g–l). These data indicate that there is a sexual difference in microglial response to elevated protein synthesis.

**Fig. 4:**
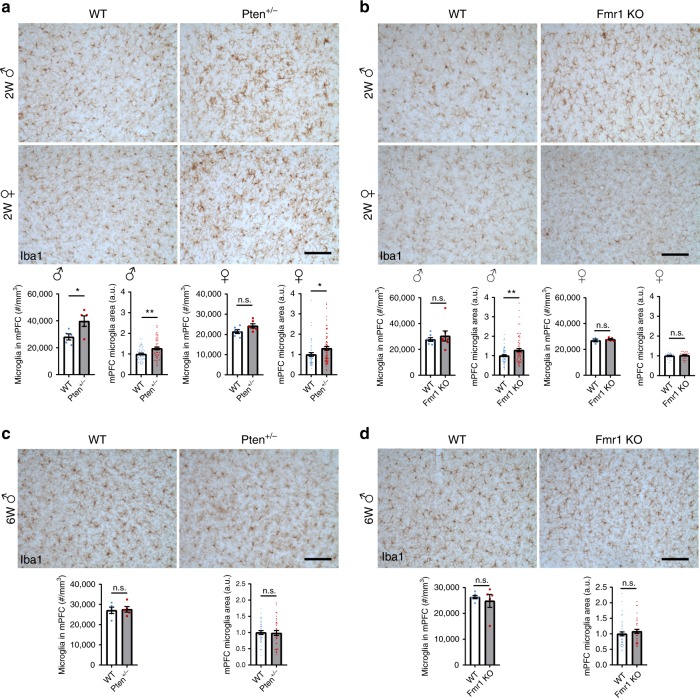
Altered microglial density and size in *Pten*^*+/−*^ and *Fmr1* KO mice. **a** Microglial density and size in the mPFC of 2-week-old male and female *Pten*^*+/-*^ mice. Male, *n* = 5 mice per genotype. Female, *n* = 6 mice per genotype. Five to ten microglia from each condition were randomly selected for measurement of cell size (cross section area). Two-sided *t*-test: male, cell density *p = 0.0317, cell size **p = 0.001; female, cell density *p* = 0.0725, cell size *p = 0.0232, n.s. not significant. Scale bar, 100 µm. **b** Microglial density and size in the mPFC of 2-week-old male and female *Fmr1* KO mice. Male, *n* = 7 mice per genotype. Female, *n* = 4 WT mice and 5 KO mice. 5-10 microglia from each condition were randomly selected for measurement of cell size (cross section area). Two-sided *t-*test: **p = 0.0046, and n.s. not significant. Scale bar, 100 µm. **c**, **d** Microglial density and size in the mPFC of 6-week-old male *Pten*^*+/−*^ (**c**) and *Fmr1* KO (**d**) mice. **c** *n* = 5 mice per condition; **d** *n* = 6 WT mice and 5 *Fmr1* KO mice. Five to ten microglia from each condition were randomly selected for measurement of cell size (cross section area). n.s., not significant by two-sided *t*-test. Scale bar, 100 μm. All data are shown as mean ± s.e.m. Source data are provided as a Source Data file.

Inactivating mutations in the *PTEN* and *FMR1* genes account for a large percentage of human syndromic ASD^3^. To determine if microglial changes observed in MG^4E^ mice also occur in mice that model these two syndromes, we measured microglial density and size in 2-week-old male *Pten*^*+/−*^ and *Fmr1* knockout (KO) mice. Microglia in the mPFC, hippocampus and striatum were significantly larger in *Pten*^*+/−*^ and *Fmr1* KO mice than WT littermates (Fig. 4a, b and Supplementary Figs. 11a, 12a). Moreover, microglial density was increased in the *Pten*^*+/−*^ mPFC and *Fmr1* KO striatum (Fig. 4a and Supplementary Fig. 12a). The lack of elevated microglial density in all brain regions of 2-week-old male *Pten*^*+/−*^ and *Fmr1* KO mice could be due to a smaller degree of protein synthesis increase relative to MG^4E^ mice. We notice that microglial density in tamoxifen-treated male *R26*^*Eif4e/Eif4e*^ (control) mice is lower than that in untreated male WT mice at 2 weeks of age (compare Figs. 3c, 4a, b), which is possibly due to growth retardation of mice caused by tamoxifen injection at P0.

The microglial morphological phenotype observed in 2-week-old male *Pten*^*+/−*^ and *Fmr1* KO mice also displays sexual dimorphism. While microglia in 2-week-old female *Pten*^*+/−*^ mice show some morphological alterations, the phenotype is not as prominent as observed in male *Pten*^*+/−*^ littermates. Compared to control mice, microglia in 2-week-old female *Pten*^*+/−*^ mice were larger in the mPFC and the striatum but not in the hippocampus, and their density was not significantly altered in all the three brain regions (Fig. 4a and Supplementary Fig. 11b). Furthermore, the density and size of microglia was normal in 2-week-old female *Fmr1* KO mice (Fig. 4b and Supplementary Fig. 12b). Similar to what was observed in MG^4E^ mice, the morphological phenotype in male *Pten*^*+/−*^ and *Fmr1* KO mice is attenuated with age, so that the phenotype in these mice disappeared by 6 weeks of age (Fig. 4c, d and Supplementary Figs. 13, 14).

### Synaptic alterations in male MG^4E^ mice

**Fig. 5:**
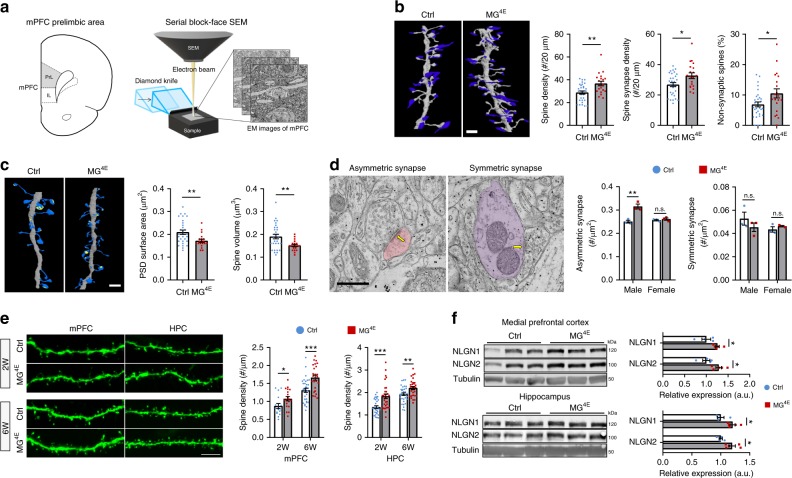
Structural and functional alterations in synapses of MG^4E^ mice. **a** Schematic diagram of mPFC serial block-face scanning electron microscopy (SB-SEM). **b** Spine density in the mPFC of 6-week-old male control and MG^4E^ mice. Representative dendrites reconstructed from SB-SEM images show dendritic spines (gray) and presynaptic terminals (blue). Graphs show spine density, synaptic spine density and percentage of non-synaptic spines. 5 control mice, 40 dendrites and 1187 spines; 6 MG^4E^ mice, 30 dendrites and 1120 spines. Two-sided *t*-test (spine density, **p = 0.0017; synaptic spine density, *p = 0.0112; non-synaptic spine *p = 0.0253). Scale bar, 2 µm. **c** Spine volume and synaptic size. Representative dendrites reconstructed from SB-SEM images show dendritic spines (blue) and postsynaptic density (PSD, light yellow). Graphs show PSD size and spine volume. 5 control mice, 40 dendrites and 1098 spines; 6 MG^4E^ mice, 30 dendrites and 999 spines. Two-sided *t* test (PSD area, **p = 0.0044; spine volume, **p = 0.0037). Scale bar, 2 µm. **d** Density of asymmetric and symmetric synapses in the mPFC of 6-week-old male and female control and MG^4E^ mice. Images show representative asymmetric and symmetric synapses (arrows). *n* = 3 mice and 30 images per mouse for each group. **p = 0.0059 by two-sided *t*-test. Scale bar, 2 µm. **e** Spine density in mPFC layer 5 neurons and hippocampal CA1 neurons of 2-week-old (2 W) and 6-week-old (6 W) male control and MG^4E^ mice. 2 W, 6 mice per genotype; 6 W, 6 control mice and 7 MG^4E^ mice; 2–5 neurons in each brain region per mouse. *p = 0.0362, **p = 0.0012 and ***p < 0.001 by two-sided *t*-test. Scale bar, 5 µm. **f** Levels of neuroligin 1 (NLGN1) and neuroligin 2 (NLGN2) in the mPFC and hippocampus. *n* = 4 control mice and 5 MG^4E^ mice. Two-sided *t* test (mPFC, *p = 0.0409 for NLGN1 and *p = 0.0279 for NLGN2; hippocampus, *p = 0.0207 for NLGN1 and *p = 0.0229 for NLGN2). All data are shown as mean ± s.e.m. Source data are provided as a Source Data file.

As microglia play important roles in neuronal development, the observed changes in microglial number and morphology could alter synaptic development and function in male MG^4E^ mice, which then leads to ASD-like behaviors. To investigate this possibility, we examined synaptic structures in layer 2 of the mPFC prelimbic area (PrL) in 6-week-old male control and MG^4E^ mice using serial block-face scanning electron microscopy (Fig. 5a). Three-dimension reconstruction of dendrites revealed that male MG^4E^ mice had higher spine density, with contributions from both non-synaptic and synaptic spines compared to control mice (Fig. 5b). Male MG^4E^ mice had smaller spines and synapses on average (Fig. 5c). To examine synaptic maturation, we counted multiple-synapse spines (MSS) and multiple-synapse boutons (MSB), which are believed to be the structural basis of synaptic multiplicity^26^. Synaptic multiplicity increases in the hippocampus during postnatal development and is considered an indicator of synapse maturation^43^. We found that male control and MG^4E^ mice had comparable MSS and MSB density in layer 2 of the PrL (Supplementary Fig. 15a, b), suggesting that reduced synapse size in male MG^4E^ mice is not indicative of immaturity.

Because dendritic spines are the postsynaptic sites for the vast majority of excitatory synapses^44^, higher spine density indicates more excitatory synapses. Indeed, transmission electron microscopy analysis revealed more asymmetric (excitatory) synapses in the PrL of 6-week-old male, but not female, MG^4E^ mice compared to control mice (Fig. 5d). This analysis also found a normal density of symmetric (inhibitory) synapses in the PrL of both male and female MG^4E^ mice (Fig. 5d). Thus, exaggerated protein synthesis in microglia increases the number of excitatory synapses in the mPFC in male mice.

Increased spine density has been observed in ASD patients^45,46^. To determine if spine density is also increased in other brain areas of male MG^4E^ mice and at other ages, we employed the Thy1-GFP transgene^47^ to label isolated neurons in 2-week and 6-week-old mice. We found that male, but not female, MG^4E^ mice had higher spine density in pyramidal neurons of mPFC layer 5 and hippocampal CA1 area at both ages (Fig. 5e and Supplementary Fig. 16a). These results indicate that elevated microglial protein synthesis increases the density of excitatory synapses in multiple brain areas.

We next examined whether levels of synaptic proteins are altered in the mPFC and hippocampus of MG^4E^ mice. We found that adult male and female control and MG^4E^ mice had comparable levels of presynaptic synaptophysin and postsynaptic neuroligin 3, neuroligin 4, PSD95 and GluA1 in the hippocampus and mPFC (Supplementary Figs. 15c, 15d, 16b, 16c). Neuroligins are cell-adhesion molecules important for synaptogenesis and have been implicated in ASD^48^. Levels of neuroligins 1–4 are increased in the hippocampus of *Eif4ebp2* knockout and transgenic βT-Eif4e mice, which was interpreted as a result of increased mRNA translation in neurons^16^. Interestingly, levels of neuroligins 1 and 2 in the hippocampus and mPFC were significantly higher in male, but not female, MG^4E^ mice, compared with sex-matched control mice (Fig. 5f and Supplementary Fig. 16b, c). This finding suggests that the observed increase in hippocampal levels of neuroligins in *Eif4ebp2* knockout and transgenic βT-Eif4e mice could be a result of microglial alterations rather than increased eIF4E activity in neurons. This argument is supported by the observation that levels of neuroligins1–4 were comparable between male control mice and male NN^4E^ mice that overexpress eIF4E in neurons (Supplementary Fig. 17). The increase in levels of neuroligins 1 and 2 is likely due to enhanced transcription in neurons, as levels of mRNAs for the two proteins show a trend of increase in male MG^4E^ mice (Supplementary Fig. 15e).

**Fig. 6:**
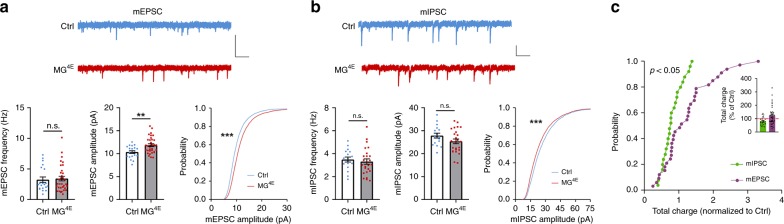
Abnormal synaptic function in male MG^4E^ mice. **a** mEPSCs recorded in mPFC layer 5 neurons of male control and MG^4E^ mice at 6–7 weeks of age. *n* = 20 cells from five control mice and 33 cells from 8 MG^4E^ mice. Two-sided *t*-test for frequency and amplitude: **p = 0.0022; n.s. not significant. Two-sided Kolmogorov–Smirnov test for cumulative probability of mEPSC amplitude: ***p < 0.001. Scale bars, 50 pA (vertical) and 0.5 s (horizontal). **b** mIPSCs recorded in mPFC layer 5 neurons of male control and MG^4E^ mice at 6–7 weeks of age. n = 17 cells from 4 control mice and 25 cells from 6 MG^4E^ mice. Two-sided *t*-test for frequency (*p* = 0.6053) and amplitude (*p* = 0.1097): n.s. not significant. Two-sided Kolmogorov–Smirnov test for cumulative probability of mIPSC amplitude: ***p < 0.001. Scale bars, 25 pA (vertical) and 0.5 s (horizontal). **c** Relative changes in mEPSC (*n* = 33 cells) and mIPSC (*n* = 25 cells) total charge transfer in MG^4E^ mice, normalized to control mice. *p* = 0.0453 by two-sided Kolmogorov–Smirnov test. All data are shown as mean ± s.e.m. Source data are provided as a Source Data file.

To investigate if alterations in synaptic structure and proteins lead to abnormal synaptic function in male MG^4E^ mice, we recorded miniature excitatory postsynaptic currents (mEPSCs) and miniature inhibitory postsynaptic currents (mIPSCs) in layer 5 pyramidal neurons of the mPFC. The amplitude, but not the frequency, of mEPSCs was increased in male MG^4E^ mice relative to control mice (Fig. 6a). In contrast, the amplitude of mIPSCs were slightly reduced in male MG^4E^ mice, as indicated by a significant but small left shift of its cumulative curve (Fig. 6b). These results suggest an alteration in the excitation/inhibition (E/I) balance, which is believed to contribute to ASD^3^, in the mPFC. Indeed, we found that the normalized total charge transfer in male MG^4E^ mice relative to control mice was significantly larger for mEPSCs than for mIPSCs (Fig. 6c). As expected on the basis of synapse density and levels of synaptic proteins, female control and MG^4E^ mice had comparable mEPSCs, mIPSCs and charge transfer in the mPFC (Supplementary Fig. 16d, e).

Collectively, our microscopic, biochemical and electrophysiological analyses reveal that elevated protein synthesis in microglia leads to synaptic alterations that are associated with ASD in male mice only, including increased spine density, increased levels of neuroligins and E/I imbalance. These synaptic changes likely contribute to deficits in social interaction, repetitive behaviors, and cognitive impairments in male MG^4E^ mice.

### Microglia are out of homeostatic state in male MG^4E^ mice

**Fig. 7:**
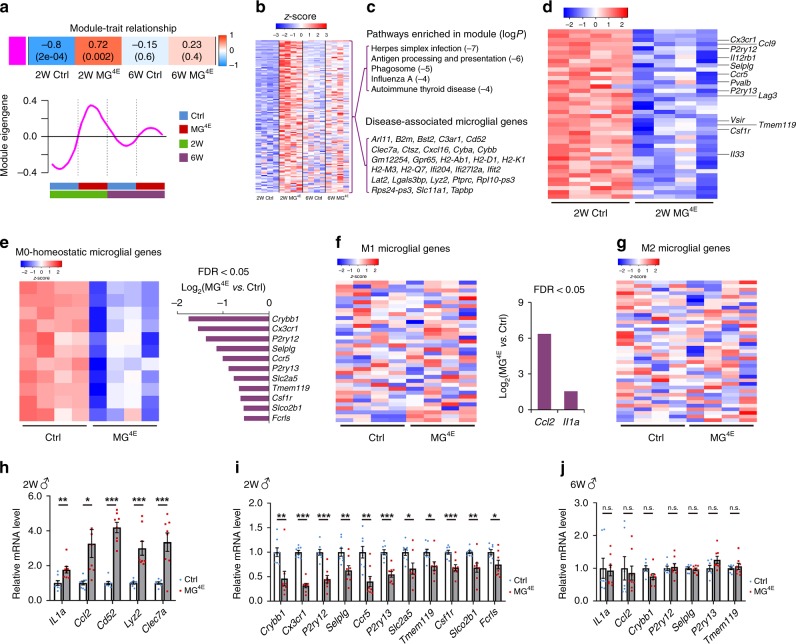
Gene expression profiles in male control and MG^4E^ mice. RNA-seq was performed using RNA samples isolated from hippocampi of 2-week-old (2 W) and 6-week-old (6 W) male control (Ctrl) and MG^4E^ mice. **a** (top) Heatmap showing module-trait relationship of magenta module. Correlation coefficients between a module eigengene and trait (coded from −1 to 1) and corresponding *p* values (two-sided Student asymptotic *p* value for a given correlation was calculated using corPvalueStudent in WGCNA package) are shown in each cell. The color indicates the level of correlation. (bottom) Plot showing the expression of the module eigengene across sample categories. **b** Heatmaps of genes (138 genes) within magenta module. **c** Selected KEGG pathways and disease-associated microglial genes enriched in magenta module (log*P* values adjusted with Benjamini correction). **d** Heatmaps of downregulated genes (FDR < 0.05) in 2-week-old MG^4E^ mice. (e Heatmaps of downregulated M0-homeostatic microglial genes in 2-week-old MG^4E^ mice. **f**, **g** Heatmaps of top 40 M1 (**f**) and M2 (**g**) microglial genes in 2-week-old MG^4E^ mice (Ctrl and MG^4E^, *n* = 4 mice). **h**, **i** RT-PCR validation of selected upregulated (**h**) and down-regulated (**i**) genes in hippocampi of 2-week-old male MG^4E^ mice. *n* = 8 per genotype. *p < 0.05, **p < 0.01 and ***p < 0.001 by two-sided *t*-test. **j** RT–qPCR validation of selected genes in hippocampi of 6-week-old male MG^4E^ mice. *n* = 7 control mice and 8 MG^4E^ mice. n.s. not significant by two-sided *t*-test. All data are shown as mean ± s.e.m. Source data are provided as a Source Data file.

To understand how microglial eIF4E overexpression alters synaptic structure and function, we analyzed hippocampal gene expression in male control and MG^4E^ mice at 2 and 6 weeks of age using RNA-Seq. Weighted gene correlation network analysis (WGCNA)^49^ revealed 11 modules of highly co-expressed genes in control and MG^4E^ mice (Supplementary Fig. 18a). Of note, the magenta module displayed a set of transcripts that were significantly upregulated in 2-week-old MG^4E^ mice (Fig. 7a). Pathway enrichment analysis of genes within the magenta module revealed top KEGG pathways related to antimicrobial activities, including herpes simplex infection, antigen processing and presentation, phagosome, influenza A and autoimmune thyroid disease (Fig. 7b, c). Some of the disease-associated microglial genes found in neurodegenerative conditions^50^ were also upregulated in 2-week-old MG^4E^ mice (28 out of 138 genes in the magenta module; Fig. 7c). By 6 weeks of age, the magenta module eigengene was no longer significantly upregulated in MG^4E^ mice, suggesting that this set of transcripts were only upregulated during postnatal development. Interestingly, the herpes simplex virus infection and autoimmune thyroid disease pathways in the magenta module have been reported to be risk factors for ASD^51–53^. In addition to the upregulated genes (Supplementary Fig. 18b, c), we identified a group of downregulated genes by comparing 2-week-old male MG^4E^ mice to control littermates (Fig. 7d and Supplementary Fig. 18b, d). Many of the downregulated genes (11/40, or 27.5%) are markers for homeostatic (M0) microglia^54^, including *P2ry12*, *P2ry13*, *Cx3cr1*, *Tmem119*, and *Slco2b1* (Fig. 7e).

In addition to M0 state, microglia can achieve two other polarized states (M1 and M2) that are characterized by specific molecular signatures^54,55^. It is proposed that M1 microglia produce pro-inflammatory cytokines and M2 microglia are anti-inflammatory^55^. We found that very few M1 and M2 markers were upregulated in 2-week-old male MG^4E^ mice (Fig. 7f, g). This indicates that elevated protein translation does not move microglia to M1 or M2 polarized states.

We validated the RNA-Seq results by measuring mRNA levels of a few select genes in 2-week and 6-week-old control and MG^4E^ mice using quantitative RT-PCR (Fig. 7h–j). The RT-PCR analysis also found that the observed gene expression changes were absent in 2-week-old female MG^4E^ mice (Supplementary Fig. 18e). Taken together, the RNA-Seq and RT-PCR analyses show that elevated protein synthesis shifts microglia from the homeostatic state to a functional state with properties that have been observed in neurodegenerative conditions.

### Male MG^4E^ microglia with altered phagocytosis and motility

**Fig. 8:**
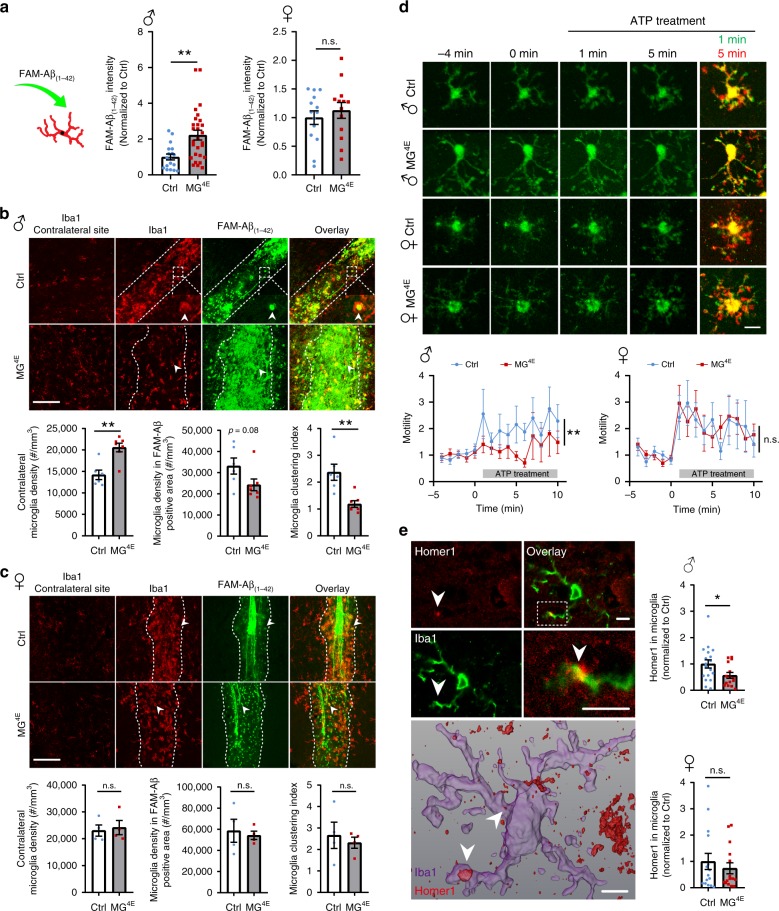
Microglial surveillance and synapse engulfment in MG^4E^ mice. **a** Phagocytosis of FAM-Aβ_(1–42)_ in cultured control and MG^4E^ microglia. Male, *n* = 18 for control and *n* = 28 for MG^4E^; female, *n* = 14 for control and *n* = 13 for MG^4E^. **p = 0.0017 and n.s. not significant (*p* = 0.4919) by two-sided *t*-test. **b**, **c** Migration of microglia into FAM-Aβ_(1–42)_ injection sites in 2-week-old male (**b**) and female (**c**) control and MG^4E^ mice. Microglia clustering index is defined as (density of Iba1^+^ cells in the FAM-Aβ-covered area)/(density of Iba1^+^ cells in a contralateral site). Male, *n* = 6 control mice and 7 MG^4E^ mice; female, *n* = 4 per genotype. Two-sided *t*-test: male mice, contralateral microglia density, **p = 0.0013; clustering index, **p = 0.0026, n.s. not significant. Scale bars, 100 μm. **d** Microglial surveillance in response to ATP treatment in male and female MG^4E^ microglia at P14–P18. Microglial baseline motility was recorded for 5 min, then ATP was bath-applied to brain slices and recorded for another 10 min. Data was normalized to the mean process motility of first 5 min. Male, *n* = 15 microglia from 3 control mice and 20 microglia from 3 MG^4E^ mice; female, *n* = 18 microglia from 3 control mice and 26 microglia from 3 MG^4E^ mice. Male, two-way ANOVA for genotype during ATP treatment, *F*_(1, 330)_ = 9.566, *p* = 0.002; female, two-way ANOVA for genotype during ATP treatment, *F*_(1, 420)_ = 0.045, *p* = 0.831. Scale bar, 10 μm. **e** Engulfment of Homer1 by microglia. Upper panel shows confocal images of Homer1 and Iba1 double immunohistochemistry, and lower panel shows 3-D reconstruction of a microglial cell and Homer1 immunoreactivity. Arrowheads denote Homer1 inside the microglia. Male, 18 microglia from 6 control mice and 15 microglia from 5 MG^4E^ mice; female, 15 microglia from 5 mice per genotype. *p = 0.0378 and n.s., not significant (*p* = 0.4891) by two-sided *t*-test. Scale bars, 5 μm. All data are shown as mean ± s.e.m. Source data are provided as a Source Data file.

Microglia are the primary phagocytes in the brain that engulf invading pathogens and cellular debris^56^. Upregulation of antimicrobial pathways including the phagosome pathway (Fig. 7c) prompted us to hypothesize that the phagocytic capacity of microglia is enhanced in 2-week-old male MG^4E^ mice. Indeed, purified microglia from male MG^4E^ mice phagocytized more than twice as many Aβ_(1–42)_ aggregates as those from male control mice, but microglia from female control and MG^4E^ mice had comparable phagocytosis (Fig. 8a).

Given that microglia have been shown to prune synapses through phagocytosis during postnatal development^23,24^, we were puzzled by how elevated spine density (Fig. 5b, e) and enhanced microglial phagocytosis (Fig. 8a) co-exist in male MG^4E^ mice. We noticed that the downregulated genes are associated with leukocyte cell–cell adhesion, leukocyte migration, and cell–cell adhesion (Supplementary Fig. 18d). Thus, we examined migration of microglia to an injection site of Aβ_(1–42)_ aggregates in 2-week-old male control and MG^4E^ mice. We found that the number of microglia migrated to the Aβ_(1–42)_ injection site was significantly reduced in male, but not female, MG^4E^ mice relative to control mice (Fig. 8b, c). These results indicate that elevated protein synthesis impairs the motility of microglia in male mice.

Microglial processes display two modes of motility: constant extension and retraction to survey the brain (baseline motility) and extension to the source of attractants such as ATP released from a damaged site (induced motility)^57^. Because synapse pruning involves contacts of microglial processes with synapses^58^, it should be affected by the motility of microglial processes. As elevated protein synthesis impaired the motility of whole microglia, it could also affect the motility of microglial processes. We monitored the baseline motility and ATP-induced motility of microglial processes in organotypic hippocampal cultures established from P14-P18 control and MG^4E^ mice harboring the CD68-EGFP transgene^59^ which is selectively expressed in microglia. Irrespective of sex, microglial processes in control and MG^4E^ mice had comparable baseline processes motility (Supplementary Fig. 18f). However, ATP-induced motility was impaired in male, but not female, MG^4E^ mice (Fig. 8d).

Synapses to be eliminated may release a factor to initiate their interactions with microglial processes, and thereby deficits in the induced motility in male MG^4E^ mice would impair engulfment of synapses. We assessed synapse engulfment by measuring the immunoreactivity of postsynaptic protein Homer1 inside microglia. Three-D reconstruction of Iba1 and Homer1 immunoreactivities revealed that the volume of Homer1 immunoreactivity inside microglia was significantly reduced in male, but not female, MG^4E^ mice relative to control mice (Fig. 8e).

Collectively, these results indicate that induced motility of microglia is impaired in male MG^4E^ mice, which might lead to reduced microglial engulfment of synapses and subsequently increased density of excitatory synapses, despite of enhanced microglial phagocytosis capacity in these mice.

## Discussion

Our results indicate that elevated neuronal protein synthesis does not lead to deficits in social interaction, but it produces some ASD-related behaviors. NN^4E^ mice of both sexes displayed elevated anxiety, a common ASD comorbidities^4^. In addition, as revealed by marble burying tests, elevated neuronal protein synthesis increased repetitive behavior in female but not male mice. These behavioral phenotypes are interesting, given the observation that elevated microglial protein synthesis impaired social behaviors without elevating anxiety in male mice. These results indicate that different ASD-like and related behaviors have distinct cellular basis. They also suggest that ASD manifestations could be different in females and males.

How does elevated protein synthesis in microglia lead to ASD-like synaptic and behavioral aberrations only in male mice? Our results indicate that the key mechanism is a sex-dependent response of microglia to elevated protein synthesis. Male microglia alter their transcriptome, which leads to changes in their function and morphology, but female microglia do not. Our transcriptomic analysis reveals that eIF4E overexpression shifts microglia from the homeostatic state to an undefined functional state only in male mice. The alterations in male microglia should result from elevated translation of some mRNAs in-these cells. However, our data do not exclude a remote possibility that elevated translation in peripheral immune cells impacts microglia differently in males and females through circulating factors, because monocytes and peripheral macrophages do overexpress eIF4E in MG^4E^ mice in the first few postnatal weeks.

Homeostatic microglia are constantly surveying their microenvironment with extremely motile processes and their processes rapidly converge at the site of injury in the brain^60,61^. The motility and convergence of microglial processes in response to ATP released from damaged cells are dependent on the purinergic receptors such as P2Y12^61,62^. It is likely that homeostatic microglia employ a similar mechanism to reach synapses tagged for elimination. We found that eIF4E overexpression downregulates many genes specific to homeostatic microglia including genes for purinergic receptors (*P2ry12* and *P2ry13*)^54^ in young male mice. As this gene expression change would predict, eIF4E overexpression dramatically diminishes the induced motility of microglia. The loss of the induced motility would impair the ability of microglia to find synapses tagged for elimination so that microglia could not efficiently prune synapses. Indeed, we found that microglia in male MG^4E^ pups contain a lower amount of engulfed Homer1 than those in control pups. This could be the main reason for observed increases in spine density and synapse density in male MG^4E^ mice. This conclusion does not exclude the possibility that elevated microglial protein synthesis increases the rate of spine formation in male mice. We found that microglia in male MG^4E^ mice have more processes relative to control mice, which would increase points of microglial contacts with neurons. The contact has been found to induce synapse formation in the cortex^63^. It would be important to identify the proteins that lead to alterations in transcription, morphology, and function in MG^4E^ microglia when their synthesis is elevated and to understand why elevated synthesis of the proteins only affects male microglia in future studies.

Our RNA-Seq data indicate that microglia in male MG^4E^ mice upregulate expression of several cytokines, including C-X-C motif chemokine 10 (CXCL10), CXCL16, C-C motif chemokine 12 (CCL12) and interleukin 1 alpha (Il1α). We speculate that these cytokines could upregulate the expression of neuroligins and modify the function of glutamate receptors via posttranslational modifications, leading to the observed increase in the amplitude of mEPSCs. The increase in the function of excitatory synapses, along with increased density of excitatory synapses, would lead to E/I imbalance. Deficits in synapse pruning would lead to the presence of excess, imprecise and inefficient neuronal connections. Both E/I imbalance and synapse pruning impairment have been linked to ASD^3^ and could be responsible for deficits in social interaction and repetitive behaviors in male MG^4E^ mice.

It is generally believed that *PTEN* haploinsufficiency elevates protein synthesis by increasing mTORC1 activity^3^. However, a recent study shows that genetic ablation of mTOR complex 2 (mTORC2), but not mTORC1, activity reverses the pathophysiology of *Pten*^+/−^ mice^64^. It is likely that the complete ablation of mTORC1 activity is not the best way to reverse mTORC1 hyperactivity in *Pten*^+/−^ mice. It is known that mTORC2 can fully activate Akt via Ser473 phosphorylation^65^, thereby increasing the activity of mTORC1, an Akt downstream target. Therefore, it could be a good strategy to treat ASD associated with elevated protein synthesis by targeting mTORC2 to reduce mTORC1 activity. There are conflicting reports on the effect of elevated protein synthesis on basal synaptic transmission. Enhanced mTORC1 activity via expression of a constitutively active form of Rheb was reported to reduce the frequency of spontaneous EPSCs in pyramidal neurons of the anterior cingulate cortex^66^. However, increases in both the frequency and the amplitude of mEPSCs were found in the hippocampal CA1 region of *Eif4ebp2* knockout mice, where mRNA translation is elevated^16,67^. The discrepancy could result from differences in the extent of translation enhancement, brain region, and recorded synaptic property in these studies. We found that elevated protein synthesis in microglia via eIF4E overexpression increases the amplitude of mEPSCs in the mPFC without affecting the frequency of mEPSCs. It would be interesting to investigate if elevated protein synthesis in multiple types of brain cells is required to affect both frequency and amplitude of mEPSCs.

Transcriptomic analyses of post-mortem brains find that genes associated with activated microglia are upregulated in the cortex of ASD patients^68,69^. Furthermore, knockdown of the microglia-specific *Cx3cr1* gene leads to a transient reduction in microglia, deficits in synapse pruning and ASD-like behaviors^26^. These findings provide some evidence for involvement of microglia in ASD; however, ASD-associated causal genetic variants in microglial genes have not been identified yet. Protein synthesis is elevated as a result of ASD-causing mutations in genes encoding negative translation regulators (such as in *PTEN*, *TSC1/2*, and *FMR1*)^12,13,15^. Our results show that elevating protein synthesis in microglia leads to ASD-like synaptic and behavioral aberrations in mice. Therefore, our study suggests that ASD-associated mutations in ubiquitously expressed genes could lead to ASD through their primary effects on microglia. In addition, our findings may provide some insights into the pathophysiology of ASD associated with other risk factors. Environmental insults, such as microbial infection during pregnancy, lead to the emergence of core ASD symptoms in the adult offspring, especially in males^70–73^. Because microglia are at the interface between the brain and environment, our findings suggest that environmental insults could increase the risk of ASD by altering functional states of microglia in the offspring. We propose that augmented responses of microglia to biochemical perturbations induced by gene mutations or microbial infection are an important underlying pathogenesis for ASD. Elucidation of the sexual difference in microglial response to the perturbations in future studies could provide innovative strategies for prevention and treatment of ASD.

## Methods

### Animals

Syn1-Cre (Stock No: 003966), GFAP-Cre (Stock No: 024098), Thy1-GFP (Stock No: 007788), *Rosa26*^*Ai9/+*^ (Stock No: 007909), *Fmr1* knockout (Stock No: 003025), CD68-EGFP (Stock No: 026827) and *Cx3cr1*^*CreER/+*^ (Stock No: 021160) mouse strains were obtained from the Jackson Laboratory. The *Pten*^*+/−*^ strain was from the National Cancer Institute. All mice were maintained at 22 °C on a 12-h/12-h light/dark cycle with ad libitum access to water and food. Animal procedures were approved by the Scripps Florida Institutional Animal Care and Use Committee.

### Generation of the *R26*^*Eif4e*^ mouse strain

To generate a transgenic mouse strain overexpressing eIF4E from the *Rosa26* locus, we generated a targeting construct by replacing the tdTomato coding sequence with the *Eif4e* coding sequence in the Ai9 construct^35^ (a gift from Hongkui Zeng, Addgene plasmid # 22799). To distinguish between endogenous and overexpressed eIF4E, we inserted a DNA sequence for the Myc tag (gccGAACAAAAACTCATCTCAGAAGAGGATCTGaatagctag) immediately before the stop code of the eIF4E coding sequence. The targeting vector was linearized and transfected into C57BL/6 embryonic stem cells. G418-resistant ES clones were screened with Southern blots. Two positive ES clones were injected into blastocysts to obtain chimeric mice. The mouse strain was maintained on the C57BL/6 background.

### Immunohistochemistry and cell counting

Mice were deeply anaesthetized with avertin and transcardially perfused with phosphate-buffered saline (PBS) and 4% paraformaldehyde (PFA) sequentially. Brains were removed, post-fixed in 4% PFA for 6 h, and then cryoprotected in 30% sucrose until sectioning. Coronal brain sections (40-µm thickness) were obtained using a sliding microtome. Brain sections were incubated with rabbit anti-Iba1 (Wako, 019-19741, 1:500) at 4 °C overnight and then biotinylated goat anti-rabbit secondary antibody (1:200, Vector, BA-1000). Sections were developed in 0.05% DAB (3,3’-diaminobenzidine) and 0.003% hydrogen peroxide in 0.1 M Tris–HCl (pH 7.5), mounted onto slides, dehydrated, and coverslipped with dibutyl phthalate-xylene mixture. Density of Iba^+^ microglia was measured under a 20× objective using Stereo Investigator software (MicroBrightField Inc.). The analysis was performed blind to genotype.

### Tamoxifen injections

Tamoxifen (Sigma, T5648) was dissolved in corn oil at a concentration of 40 mg/ml. P0 mice were subcutaneously injected with tamoxifen solution around the lower neck region one time at 180 mg/kg (~7 μl per mouse).

### Immunocytochemistry

Cultured microglia were fixed for 20 min with 4% PFA and 4% sucrose at room temperature. Cells were washed with PBS and permeabilized with 0.25% Triton X-100 in PBS. After being washed three times with PBS, microglia were incubated with a blocking buffer (PBS containing 5% BSA and 0.1% Triton X-100) for one hour at room temperature. Afterwards, cells were incubated with the following primary antibodies in the blocking buffer at 4 °C overnight: rabbit anti-Iba1 (Wako, 019-19741, 1:500), mouse anti-CD11b (Invitrogen, 12-0112-82, 1:100), rabbit anti-eIF4E (Cell Signaling Technology, #2067, 1:500) and goat anti-Iba1 (Abcam, ab5076, 1:500). Appropriate DyLight conjugated secondary antibodies were used after primary antibodies were washed off with PBS three times. Nuclei were counterstained with DAPI (Invitrogen D1306, 10 mg/ml stock solution, 1:10,000 dilution). Images were acquired using a Nikon C2+ confocal microscope.

### Three-dimensional reconstruction of microglia

Coronal sections of the mPFC from 2-week and 6-week-old mice were stained with rabbit anti-Iba1 (Wako, 019-19741, 1:500) overnight, followed by Alexa Fluor 488-conjugated (711-545-152, Jackson ImmunoResearch, 1:500) secondary staining. Images were acquired on a Nikon C2+ confocal laser scanning microscope using a 40× oil objective. Z-stack images were recorded with a 0.8-μm interval for 27 steps. 3-D images of microglia were constructed using Amira software (Thermo Fisher Scientific).

### Microglial engulfment of Homer1

Coronal brain sections from 2-week-old male and female mice were incubated with rabbit anti-Iba1 (Wako, 019-19741, 1:500) and chicken anti-Homer1 (Synaptic Systems, 160006, 1:500) overnight, followed by Alexa Fluor 594 (703-585-155, Jackson ImmunoResearch, 1:500) and 649 (711-605-152, Jackson ImmunoResearch, 1:500) conjugated secondary antibodies. Images were acquired on a Nikon C2+ confocal laser scanning microscope using a 40× oil objective, laser power and gain were kept consistent throughout the experiment. *Z*-stack images were acquired with a 0.8-μm interval for 21 steps. Images were analyzed using Amira software (Thermo Fisher Scientific) to create a 3D surface rendering of microglia with a threshold to ensure that microglial processes were accurately reconstructed. This rendering was used to mask the Homer1 channel, and the overlapped volume was considered as Homer1 engulfed by microglia.

### Western blotting

Hippocampal and prefrontal cortical tissues were dissected on ice using a coronal Brain Matrix (Roboz, SA-2175). Dissected brain tissues or cultured cells were lysed on ice for 30 min in lysis buffer containing 10 mM Tris (pH 7.4), 1% Triton X-100, 150 mM NaCl, 10% glycerol, and freshly added protease inhibitors (Roche Complete Protease Mini, #4693159001) and phosphatase inhibitors (PhosStop pellets, Sigma Aldrich, #4906845001). Lysates were centrifuged at 15,000×*g* for 30 min at 4 °C, and supernatants were saved. Protein samples were run on SDS-PAGE gels and transferred to PVDF membrane. Membrane was blocked with Odyssey Blocking Buffer (Thermo Fisher Scientific). The following primary antibodies were used: mouse anti-α-tubulin (Sigma-Aldrich T6074, 1:10,000), mouse anti-PSD95 (Thermo Fisher Scientific MA1-045; 1:1000), rabbit anti-eIF4E (Cell Signaling Technology, #2067, 1:1000), mouse anti-β-actin (Sigma-Aldrich A5441, 1:10,000), rabbit anti-GluA1 (Millipore AB1504, 1:1000), rabbit anti-Myc (Cell Signaling Technology, #2278, 1:1000), rabbit anti-synaptophysin (Invitrogen, MA5-14532, 1:1000), mouse anti-neuroligin 1 (Synaptic Systems, 129111, 1:1000), rabbit anti-neuroligin 2 (Synaptic Systems, 129202, 1:1000), mouse anti-neuroligin 3 (Synaptic Systems, 129311, 1:1000), mouse anti-neuroligin 4 (Synaptic Systems, 129403, 1:1000), mouse anti-GFAP (MA5-12023, Thermo Fisher Scientific, 1:1000), rabbit anti-P2Y12 (702516, Thermo Fisher Scientific, 1:1000), rabbit anti-eIF4G (Cell Signaling Technology, #2498, 1:1000), and mouse anti-eIF4E (sc-9976, Santa Cruz Biotechnology, 1:100). Appropriate IRDye infrared secondary antibodies (LI-COR Biosciences) were used at a dilution of 1:10,000. Odyssey Infrared Imaging System (Image Studio Lite Ver 4.0, LI-COR Biosciences) was used to detect and quantify signals of target proteins.

### eIF4E and eIF4E-Myc expressing constructs

The mouse *Eif4e* coding sequence alone or extended at its 3′ end with a sequence encoding the Myc tag (gccGAACAAAAACTCATCTCAGAAGAGGATCTGaatagctag, where the Myc-encoding sequence is listed in capital letters) was cloned into pUltra (a gift from Malcolm Moore; Addgene plasmid #24129).

### Immunoprecipitation

HEK293 cells were transfected with plasmid constructs (0.6 µg/kb) expressing eIF4E or eIF4E-Myc using lipofectamine 3000 (Invitrogen). Cells were harvested 48 h post transfection and lysed with a buffer containing: 50 mM Tris pH 7.5, 150 mM NaCl, 10% glycerol, 0.1% NP-40, 2 mM DTT and freshly added protease inhibitors (Roche Complete Protease Mini, #4693159001) and phosphatase inhibitors (PhosStop pellets, Sigma Aldrich, #4906845001). Cleared cell lysates were incubated with either anti-eIF4E antibody (A301-154A, Bethyl Laboratories, 1:100) or IgG on ice for 2 h. Protein G-Agarose (11719416001, Roche) was then added to each sample, and the mixture was incubated at 4 °C under rotary agitation for 4 h. Immunoprecipitates were washed with the buffer five times and eluted with the SDS-loading buffer for immunoblotting analysis.

### Microglial culture

Purification of primary microglia was performed using an immunopanning protocol^36^. Briefly, a mouse brain at P10 was digested with 2 ml of buffer containing 2.4 mg papain (Worthington, LS003126) and 1 mg deoxyribonuclease I (Sigma, DN25) at 37 °C for 30 min. The brain was then mechanically dissociated by gentle trituration using a 1 ml pipette. Cell suspension was transferred to a 10 cm culture dish pre-coated with rat anti-mouse CD45 antibody (Invitrogen, Cat. 17045182) and incubated for 45 min at room temperature. The culture dish was then washed with Dulbecco’s PBS 8–10 times. Culture medium (DMEM with 10% FBS, 1% GlutaMAX, 1% penicillin/streptomycin) was added to the dish, and the dish was returned to an incubator until harvest.

### Astrocyte culture

Astrocytes were isolated from brains of control and AC^4E^ mice at P3. Brains were isolated and mechanically dissociated in HBSS and papain (Worthington, LS003126) for 20 min at 37 °C. Afterwards, papain was inhibited with Dulbecco’s modified Eagle’s medium (DMEM) containing 10% FBS. Cell suspensions were subsequently plated into T75 flasks. Astrocytes were cultivated till confluency and subsequently shaken for 18 h (200 rpm). After the medium was removed, astrocytes in flasks were trypsinized and plated onto coverslips or plates for further experiments.

### Primary neuronal culture

Hippocampal neurons were cultured from P0 newborn mice^74^. Isolated hippocampi were removed and digested with 20 U/ml of papain in Hank’s Balanced Salt Solution (HBSS) at 37 °C for 30 min. Dissociated neurons were grown in Neurobasal media A (Invitrogen) supplemented with 2% B27, 1% GlutaMAX (Gibco) and 1% penicillin-streptomycin at 37 °C and 5% CO_2_ incubator.

### Tissue processing for SB-SEM

Mice were anesthetized and transcardially perfused with 2% PFA and 2.5% glutaraldehyde in 0.15 M cacodylate buffer (pH 7.4). The brains were removed and stored overnight at 4 °C in fresh perfusion solution. The brains were sliced in cold 0.15 M cacodylate buffer into 150-µm coronal sections, which were used for dissection of the prelimbic area of the mPFC. Tissue blocks were placed in cacodylate buffer containing 2% OsO4/1.5% potassium ferrocyanide for 1 h. Samples were placed in 1% thiocarbohydrazide (Ted Pella) in ddH_2_O for 20 min and then in 2% aqueous OsO4 for 30 min. Tissues were incubated in 1% uranyl acetate at 4 °C overnight and fresh lead aspartate solution at 60 °C for 30 min to enhance membrane contrast. On the next day, samples were dehydrated using an ascending series of ethanol (50, 70, 90, and 100%, 5 min each), followed by ice-cold dry acetone for 10 min. Tissues were then gradually equilibrated with Epon 812 resin (EMS, Hatfield, PA, USA). To enhance specimen conductivity^75^, samples were embedded in Epon 812 resin containing 7% (w/v) carbon black powder (a kind gift from Dr. Nobuhiko Ohno, Jichi Medical University, Tochigi-ken, Japan), mounted on aluminum rivets, and then cured in a dry-oven at 60 °C for 2 days.

### Acquisition of SB-SEM datasets and analysis

A tissue block was trimmed flat using a glass knife. Silver paste was applied to the specimen to ground the resin to the aluminum pin. The pin was then coated with 10 nm of gold-palladium in a sputter coater to further enhance conductivity. Specimens were imaged with a Merlin VP field emission scanning electron microscope (Carl Zeiss) equipped with 3View2 in-chamber ultramicrotome technology and a backscattered electron detector (Gatan). Serial images were acquired with a 30-μm aperture, high vacuum, acceleration voltage of 2.5 kV, image size of 5000 by 5000 pixels, dwell time of 3.5 µs, and X–Y resolution of 10.8 nm at a nominal thickness of 50 nm.

Eleven stacks (5 stacks for control mice, 6 stacks for MG^4E^ mice, one stack per mouse) of 500 serial images were obtained for layer 2 of the prelimbic area of the mPFC. The serial images obtained were processed with Image J and Fiji plugins TrakEM2 software (http://fiji.sc/wiki/index.php/Fiji). We randomly selected spiny dendritic segments that could be followed within the stack and whose linear lengths was at least 15 μm. To make sure that our analysis was restricted to pyramidal neurons, we avoided all dendritic segments with few or no spines. A synapse was defined by the presence of a presynaptic bouton with at least three synaptic vesicles within a 50 nm distance from the membrane facing a postsynaptic density (PSD). All dendritic spines were divided into spines with synapses and spines lacking synapses. In total, 70 dendritic branches were segmented in L2. Dendritic branches and all of their protrusions were segmented manually in the reconstruction software (https://synapseweb.clm.utexas.edu/software-0) by three trained annotators who were blind to genotype.

### Transmission EM analysis

Both male and female MG^4E^ and control mice were anesthetized and perfused with 2% PFA and 2.5% glutaraldehyde in 0.15 M cacodylate buffer (pH 7.4). The brains were sliced into 150-µm coronal sections using a vibratome and the prelimbic area of the mPFC was dissected out. Tissue blocks were prepared using standard procedures for TEM^76^. Tissues were postfixed in 2% OsO4 for 1 h, en bloc stained with 1% uranyl acetate, dehydrated, and then embedded in Epon 812 resin (EMS). Ultrathin (70-nm-thickness) sections were collected on the 200-mesh grids and post-stained with uranyl acetate followed by lead citrate. For each animal, 30 images were recorded on a Tecnai F20 TEM (FEI) (2500× magnification, 120 kV). Counting frame (6 × 6 μm^2^) was placed on each image and synapses with a PSD as well as clusters of synaptic vesicles were identified. Synapses were classified into either asymmetric (Gray’s type 1) or symmetric (Gray’s type 2) based on their distinct PSD shapes. Synaptic density values were averaged to produce animal means. All analyses were performed blind to experimental conditions.

### Stereotaxic injection of FAM-Aβ

FAM-Aβ_(1–42)_ peptides (Anaspec, AS-23525) were dissolved in trifluoroacetic acid (Sigma, T6508), lyophilized in a SpeedVac, and resuspended in PBS/DMSO solution at 37 °C overnight to a final concentration of 2 μg/μl. FAM-Aβ aggregates (500 nl) were stereotaxically injected into hippocampi of control and MG^4E^ mice at P14 using the following coordinates (relative to the bregma): AP (anterior posterior), −2.25 mm; ML (medial lateral), ±1.80 mm; DV (dorsal ventral), −2.0 mm. Mice were perfused 18–19 h after FAM-Aβ injection.

### Aβ phagocytosis assay

FAM-Aβ_(1–42)_ aggregates were added to primary microglial culture at a concentration of 1.8 μg/ml and incubated for 4 h at 37 °C. Extracellular FAM-Aβ_(1–42)_ was quenched with 0.2% trypan blue in PBS for 1 min. FAM-Aβ_(1–42)_ fluorescence signal intensity was measured at 485 nm excitation and 538 nm emission using a CLARIOstar (BMG LABTECH) microplate reader.

### Electrophysiology

Mice at P42–P49 were used for electrophysiological recording. Mice were transcardially perfused with oxygen-saturated ice-cold cutting solution containing (in mM): 124 choline chloride, 26 NaHCO_3_, 2.5 KCl, 3.3 MgCl_2_, 1.2 NaH_2_PO_4_, 0.5 CaCl_2_ and 1 d-glucose. Brains were rapidly removed and placed in ice-cold cutting solution equilibrating with 95% O_2_ and 5% CO_2_. Medial prefrontal cortical slices (300 μm) were obtained using a vibratome (Leica VT 1200S, Germany) and transferred to oxygenated artificial cerebrospinal fluid (aCSF) composed of (mM): 124 NaCl, 3 KCl, 26 NaHCO_3_, 1.25 NaH_2_PO_4_, 1 MgSO_4_, 2 CaCl_2_, and 10 d-glucose. Brain sections were recovered in aCSF at 32 °C for at least 1 h. Afterwards, a slice was gently transferred to a recording chamber (RC-27, Warner Instruments, Hamden, CT) at room temperature. The chamber was perfused with circulated oxygenated aCSF at a flow rate of 2–3 ml/min. Prelimbic layer 5 pyramidal neurons were visually identified in slices using an infrared-differential interference contrast microscope (Scientifica, UK). Whole-cell patch-clamp recordings were performed using borosilicate glass pipettes (ID: 0.68 mm, OD: 1.2 mm, WPI, Sarasota, FL) of 3–5 MΩ pulled with a micropipette puller (P-1000; Sutter Instrument, Novato, CA). For mEPSC recording, pipettes were filled with internal solution containing (in mM): 115 CsMeSO_3_, 20 CsCl, 10 Hepes, 0.6 EGTA, 4 MgATP, 0.3 Na_3_GTP, 1 QX-314, 2.5 MgCl_2_, 10 Na_2_-Phosphocreatine (pH 7.3 with CsOH, osmolarity 285 mM). For mIPSC recording, pipettes were filled with internal solution containing (in mM): 100 CsCl, 35 CsMeSO_3_, 10 Hepes, 0.5 EGTA, 4 MgATP, 0.3 Na_3_GTP, 5 QX-314, and 10 Na_2_-Phosphocreatine (pH 7.3 with CsOH, osmolarity 285 mM). Neurons were held at −70 mV in voltage-clamp mode without serious resistance and liquid junction compensation. Neurons with Ra <25 MΩ were recorded. mEPSCs were recorded in the presence of 1 μM tetrodotoxin and 100 μM picrotoxin. To isolate mIPSCs, we used 1 μM tetrodotoxin, 20 μM CNQX and 50 μM APV.

Experimenters were blind to genotype. Signals were acquired with Multiclamp 700B and Digidata 1550A (Molecular Devices, San Jose, CA). Data were low-pass filtered at 2.9 kHz and sampled at 10 kHz. mEPSCs and fEPSPs were analyzed with Clampfit 10.6 software. Detection threshold was set at 5 pA (mEPSC) or 6 pA (mIPSC), and the first 200 events were sampled per neuron. Total charge transfer was calculated by summing the charge transfer of all individual events detected over the first 2-min acquisition period for each neuron.

### SUnSET

400-μm-thick hippocampal slices were obtained using a vibratome and recovered in aCSF for 2 h at 32 °C. Puromycin (10 μg/ml) was applied to slices for 1 h to label newly synthesized proteins. To measure protein synthesis in microglia, puromycin (10 μg/ml) was applied to cultured microglia for 1 h. Tissues and cultured microglia were then lysed and used for Western blot. Mouse anti-puromycin antibody (1:2000, Millipore, MABE343) was used to detect puromycin-incorporated proteins.

### Behavioral tests

Male and female mice at 2-month-old of age were tested during the dark (active) phase of a 12/12 h reversed light-dark cycle. Mice were moved to a holding room in the behavioral testing area at least 1 h ahead of behavioral assays. Apparati were cleaned with 1% Micro-90 between each trial. Automatic scoring was performed using the Ethovision XT video tracking system (Noldus, Netherlands). Mice were tested in batteries with at least 3 days between assays. Details of these paradigms are listed below.

For open field test, a mouse was placed in the open field arena (43.8 cm × 43.8 cm × 32.8 cm) for 30 min. Total distance moved, center zone duration and velocity were recorded automatically.

For elevated plus maze test, a mouse was placed in the center of the maze to start the test. Each trial lasts for 5 min. Total distance moved and durations in open and closed arms were recorded automatically.

For light-dark box test, a mouse was placed in the light chamber and allowed to explore for 5 min. Latency to enter the dark chamber, time in the light chamber and number of crossings between chambers were automatically recorded.

Spontaneous alternation in T-maze was used to assess working memory in mice. Briefly, mice received two successive non-rewarded trials in one day. On trial one mice were allowed to enter one of the two unfamiliar arms. After staying 10 s in the chosen arm, mice were removed from the maze and placed back on the start arm for the second trial. A correct choice was to explore the arm that was not previously explored.

Mice were placed on a rotating rod (ENV-577M, Med Associates Inc.) for rotarod tests. The speed of rotation was gradually increased from 4 to 40 rpm over a 5-min period. Mice received three trials per day for two days, and each trial was spaced at least 1 h apart. The latency to fall was measured.

For fear conditioning test, a mouse was placed into a Phenotyper chamber (29.2 cm × 29 cm × 30.5 cm, Noldus) equipped with an electrified floor and speaker. Training consisted of a 150 s baseline followed by three 0.75-mA foot shocks. Mice were tested for contextual fear memory 24 h after training by returning to their training chambers for 5 min. Freezing behavior was automatically recorded by using the Ethovision XT video tracking system.

For marble burying test, mice were placed individually in a cage filled with 5 cm of corncob bedding and 20 black marbles arranged on top. The test lasted for 30 min, and the number of marbles that were at least two-thirds buried at the end of a trial were counted^38^.

For self-grooming test, mice were placed into a new cage with fresh bedding but no cardboard or nesting material. Self-grooming behavior was video recorded for 30 min and scored manually afterward. Experimenters were blind to genotype during scoring.

A three-chamber arena was used to assess sociability in mice^77^. A test mouse was placed in the central chamber and allowed to explore the empty apparatus for 5 min. Immediately after habituation, a stimulus mouse (same-sex, similar age, and unfamiliar mouse) was introduced into a wire cage located in one of the two side chambers. An identical empty wire cage was placed in the other side chamber. The test mouse was allowed to explore the arena for 10 min. Time spent in each chamber and time spent in sniffing the stimulus mouse (or empty wire cage) was recorded by Ethovision XT video tracking system. In addition to analyzing the time spent in each chamber or investigation time, we also calculated the social preference index, which represents the difference between time spent in investigating the stimulus mouse vs. object, divided by total time spent in investigating both targets.

For social recognition test, a tested mouse was housed alone for 2 h in a home cage-like environment^38^. A same sex, juvenile (3–4 weeks old) stimulus mouse was placed into an acrylic tube (7.25 cm in diameter, 12.5 cm tall) with holes at the bottom. Social recognition test consisted of 5-min stimulus presentations separated by 10-min inter-trial intervals; the first four presentations (H1–H4) were of the same stimulus mouse, with the fifth using a novel juvenile (non-littermate juvenile). Time spent in sniffing the stimulus mouse was manually scored from video. Habituation index is calculated using the formula: (H1 investigation time – H4 investigation time)/total H1 and H4 investigation time.

Novel object recognition was conducted in a three-chamber arena. A test mouse was placed in the middle chamber and allowed to explore the two side chambers with two identical objects located in each side chamber for 10 min. Immediately after this, one object was replaced with a novel object and the test mouse was allowed to explore for another 10 min. Time spent investigating the object (nose-point tracking) was automatically recorded by Ethovision XT video tracking system. Discrimination index is defined as (novel object time – familiar object time)/total time on two objects.

### RNA-Seq analysis

Total RNA was extracted from dissected hippocampal tissues using a Quick-RNA Miniprep Kit (Zymo Research, R1054). RNA was quantified using the Qubit 2.0 Fluorometer (Invitrogen, Carlsbad, CA) and run on the Agilent 2100 Bioanalyzer RNA nano chip (Agilent Technologies, Santa Clara, CA) for quality assessment. All RNA samples were of excellent quality with RNA Integrity Number (RIN) > 8.0 and were processed for mRNA_seq library preparation. The final mRNA libraries were validated on the bioanalyzer DNA chips, normalized to 1 nM, pooled equally, loaded onto the NextSeq 500 (Illumina) at a final concentration of 1.8 pM, and sequenced using 2 × 40 bp paired-end chemistry. Library preparation and sequencing was performed at the Scripps Florida Genomic Core.

Reads were trimmed off for sequencing adapter and were mapped to the mouse genome using the STAR version 2.5.2a aligner. Differential gene expression analysis was performed with genes with CPM > 0.5 in at least four samples, using Bioconductor packages edgeR^78^ with false discovery rate (FDR) threshold <0.05 (http://www.bioconductor.org). Weighted gene co-expression network analysis (WGCNA) was performed to find modules of highly correlated genes via its R package^49^. WGCNA clustering, using the “blockwiseModules” function with the network type “signed hybrid”, a power parameter of 5 (as established by scale-free topology network criteria), and minModSize = 50, minKMEtoStay = 0.3, deepSplit = 2, mergeCutHeight = 0.15, dissected the data into 11 modules. The gene set enrichment and pathway analysis were performed using online tools DAVID^79^ (https://david.ncifcrf.gov) and Generic GO Term Finder^80^.

### Quantitative reverse transcription PCR

Mouse hippocampi were collected at 2-week and 6-week of ages. Total RNA was extracted from Trizol homogenates following the manufacturer’s protocol. Following DNaseI (New England Biolabs) treatment, cDNA was synthesized using M-MuLV reverse transcriptase (New England Biolabs) with oligo dT primers. Candidate genes were then quantified by real-time PCR using SYBR Green Mix (Roche). The actin gene ACTB was used as control. The PCR primers for each gene are listed (Supplementary Table 1).

### Two-photon imaging of microglial motility and data analysis

We performed two-photon imaging to visualize microglial motility according to a previously described protocol^81^. Briefly, acute brain slices (300 µm thick) were prepared from R*26*^*Eif4e/Eif4e*^;CD68-EGFP (control) or *Cx3cr1*^*CreER/+*^;*R26*^*Eif4e/Eif4e*^;CD68-EGFP (MG^4E^) mice at P14-P18 using a vibratome (Leica VT 1200S, Germany) and transferred to oxygenated artificial cerebrospinal fluid (aCSF) composed of (mM): 124 NaCl, 3 KCl, 26 NaHCO_3_, 1.25 NaH_2_PO_4_, 1 MgSO_4_, 2 CaCl_2_, and 10 d-glucose. Brain sections were recovered in aCSF at 32 °C for 1 h. Microglia were imaged at a depth of 50–100 µm in the brain slice using a Leica TCS SP8 MP multiphoton microscope (25× lens). Stacks of 26 images with 2-µm intervals were acquired every 60 s for 5 min (base line) and for another 10 min after ATP (1 mM) was bath-applied to brain slices. Images were analyzed using ProMoIJ, an ImageJ macros that perform automatic microglial motility analysis^82^. Briefly, microglia were cropped and aligned, subsequently individual microglial processes were selected for automated analysis of their motility. Process motility was analyzed by reconstructing the process 3D skeleton and was calculated as the absolute difference of process length between two consecutive time frames. Specifically,

$${\mathrm{Process}}\,{\mathrm{motility}} = \frac{{\sqrt {({\mathrm{Length}}_{f + 1} - {\mathrm{length}}_f)^2} }}{t},$$

where *f* is time frame and *t* corresponds to the time interval between consecutive frames (1 min).

### Statistical analysis

Statistical analyses were performed using SPSS. Shapiro-Wilk (*n* < 10) and D’Agostino and Pearson omnibus (*n* > 10) normality tests were performed to determine if values fit a Gaussian distribution. Comparisons between two groups/treatments were performed using two-tailed Student’s *t*-test or Chi-square test as applicable. Cumulative curves were compared using Kolmogorov–Smirnov test. For multiple comparisons, two-way ANOVA with Fisher’s LSD post hoc test were used. The minimal level of significance was set at *p* < 0.05. Statistical parameters are detailed in the legend for each figure.

### Reporting summary

Further information on research design is available in the Nature Research Reporting Summary linked to this article.

## Supplementary information

Supplementary Information

Peer Review File

Reporting Summary

## Data Availability

RNA-seq data have been deposited to the Sequence Read Archive (SRA) at NCBI and are available at the accession number PRJNA609402 [https://www.ncbi.nlm.nih.gov/sra/?term=PRJNA609402]. The source data underlying Figs. 1b–g, 2b–j, 3c, f, g, j, m, n, 4a–d, 5b–f, 6a–c, 7h–j, and 8a–e and Supplementary Figs. 1–5 and 7–18 are provided as a Source Data file.

## Source data

Source Data
