# Supplementary material for: Elevated protein synthesis in microglia causes autism-like synaptic and behavioral aberrations
Source: Nat Commun. 2020 Apr 14;11:1797. doi: 10.1038/s41467-020-15530-3 (PMC7156673; doi:10.1038/s41467-020-15530-3)
Supplement: Supplementary file 1 — Supplementary Information [file 41467_2020_15530_MOESM1_ESM.pdf]

Elevated protein synthesis in microglia causes autism-like synaptic and behavioral aberrations

Xu et al.

SUPPLEMENTARY FIGURES

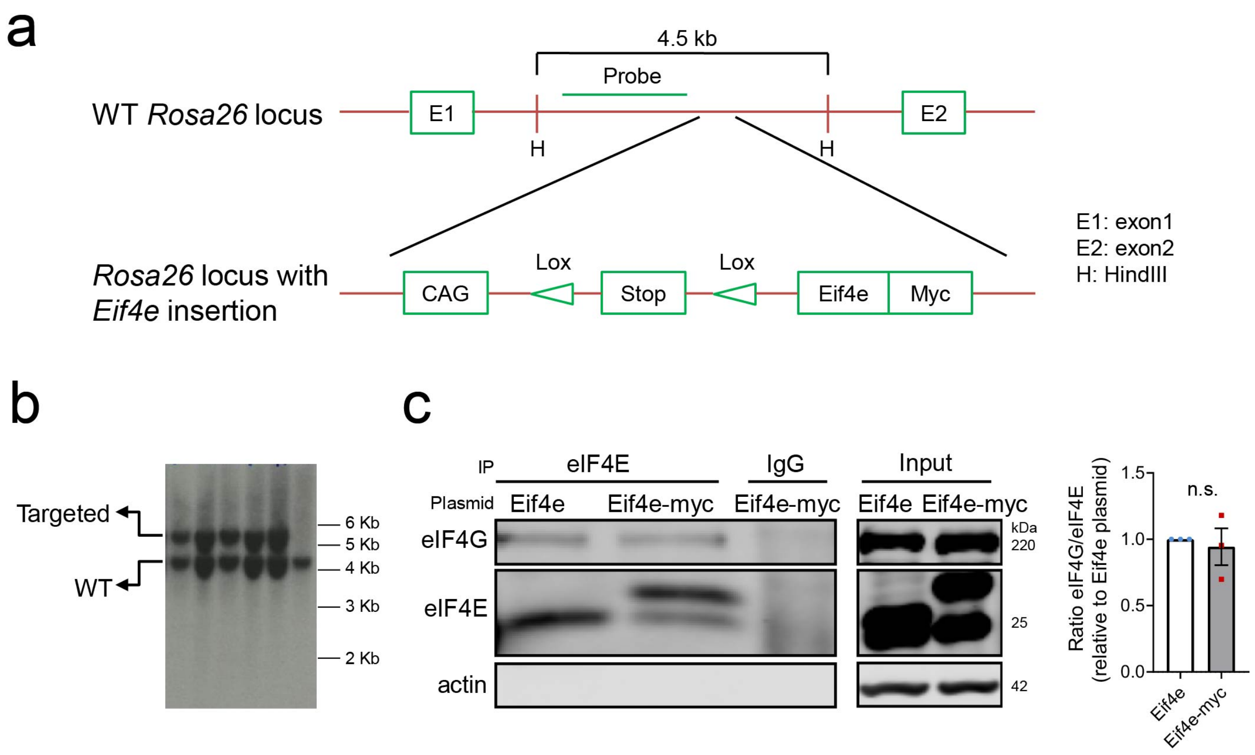

Supplementary Fig. 1. Generation of a *R26*^{Eif4e} mouse line. a, Schematic diagram of the gene targeting strategy for inserting the *Eif4e-Myc* cassette into the *Rosa26* intron between exons 1 and 2. **b**, Southern blot screening of HindIII-digested genomic DNA isolated from ES cells. WT band, 4.5 kb; targeted band, 5.6 kb. **c**, eIF4E-eIF4G interactions revealed by eIF4E immunoprecipitation (IP). Protein extracts were prepared from HEK293 cells transfected with either an Eif4e or an Eif4e-myc expression construct. n = 3 per conditions. n.s., not significant (p = 0.7053) by two-sided *t* test. All data are shown as mean ± s.e.m. Source data are provided as a Source Data file.

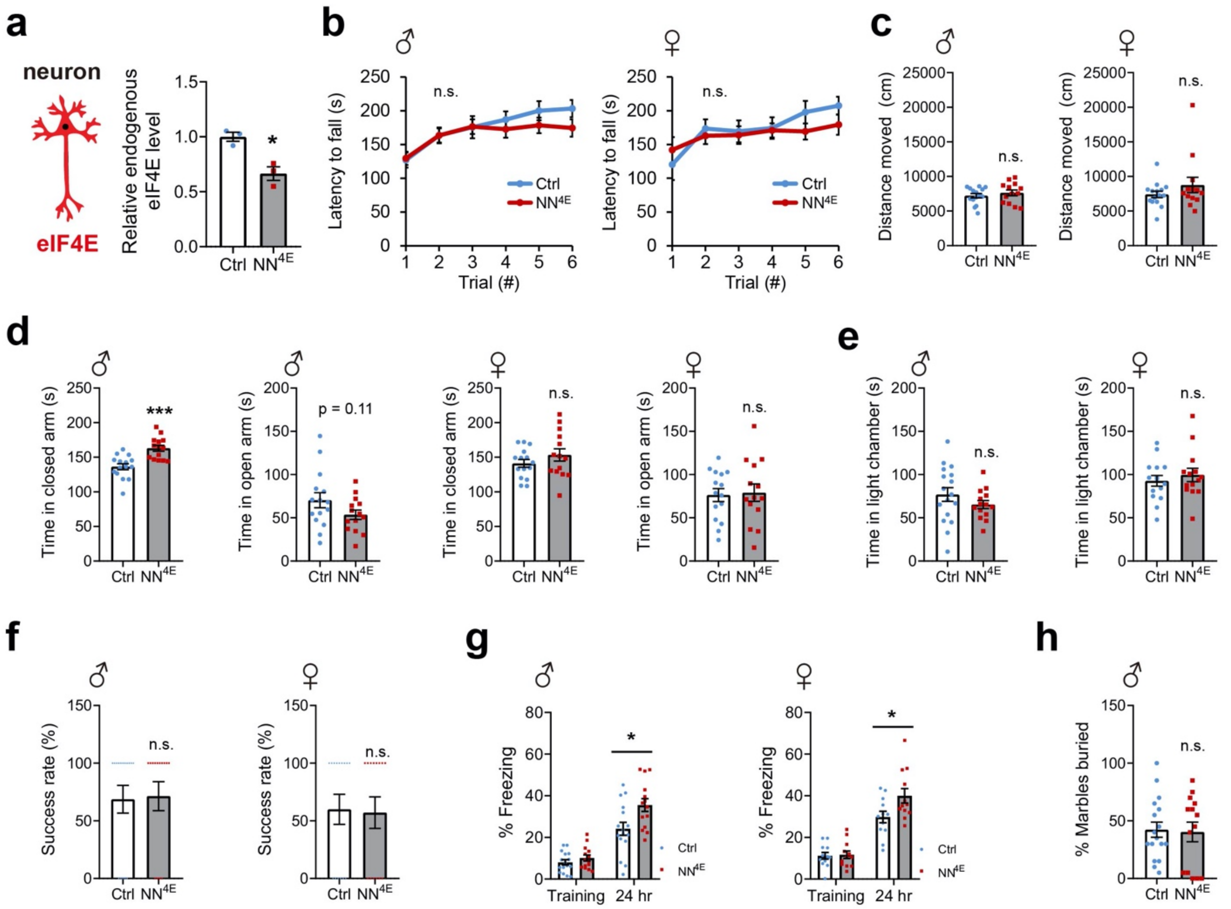

Supplementary Fig. 2. Behaviors of mice overexpressing eIF4E in neurons (NN^{4E}). a, Overexpression of eIF4E-Myc decreased endogenous eIF4E levels in the hippocampus [1.00 ± 0.12 for control (Ctrl) vs. 0.60 ± 0.02 for NN^{4E}]. n = 3 per genotype; *p = 0.0107 by two-sided *t* test. **b**, Male and female NN^{4E} mice displayed comparable performance in accelerating rotarod rod tests. Male: n = 17 control mice and 14 NN^{4E} mice; Female: n = 14 control mice and 13 NN^{4E} mice. n.s., not significant by two-way ANOVA. **c**, Total distance moved over 30 min in open field tests. Male: n = 15 control mice and 13 NN^{4E} mice; Female: n = 15 control mice and 13 NN^{4E} mice. n.s., not significant by two-sided *t* test. **d**, Time spent in the closed arm and open arm in elevated plus maze tests. Male: n = 15 control mice and 14 NN^{4E} mice; Female: n = 15 control mice and 14 NN^{4E} mice. Two-sided *t* test: ***p = 0.0002; n.s., not significant. **e**, Time spent in light chamber in light-dark box tests. Male: n = 17 control mice and 14 NN^{4E} mice; Female: n = 15 control mice and 14 NN^{4E} mice. n.s., not significant by two-sided *t* test. **f**, Working memory revealed by T-maze alternation tests. Male: n = 16 control mice and 14 NN^{4E} mice; Female: n = 15 control mice and 14 NN^{4E} mice. n.s., not significant by two-sided Chi-square test. **g**, Enhancement of contextual fear memory in male and female NN^{4E} mice. Male: n = 16 control mice and 14 NN^{4E} mice; Female: n = 12 control mice and 12 NN^{4E} mice. Two-sided *t* test: male, *p = 0.0146; female, *p = 0.0310. **h**, Percentage of marbles buried by male NN^{4E} mice. n = 17 control mice and 14 NN^{4E} mice. n.s., not significant by two-sided *t* test. All data are shown as mean ± s.e.m. Source data are provided as a Source Data file.

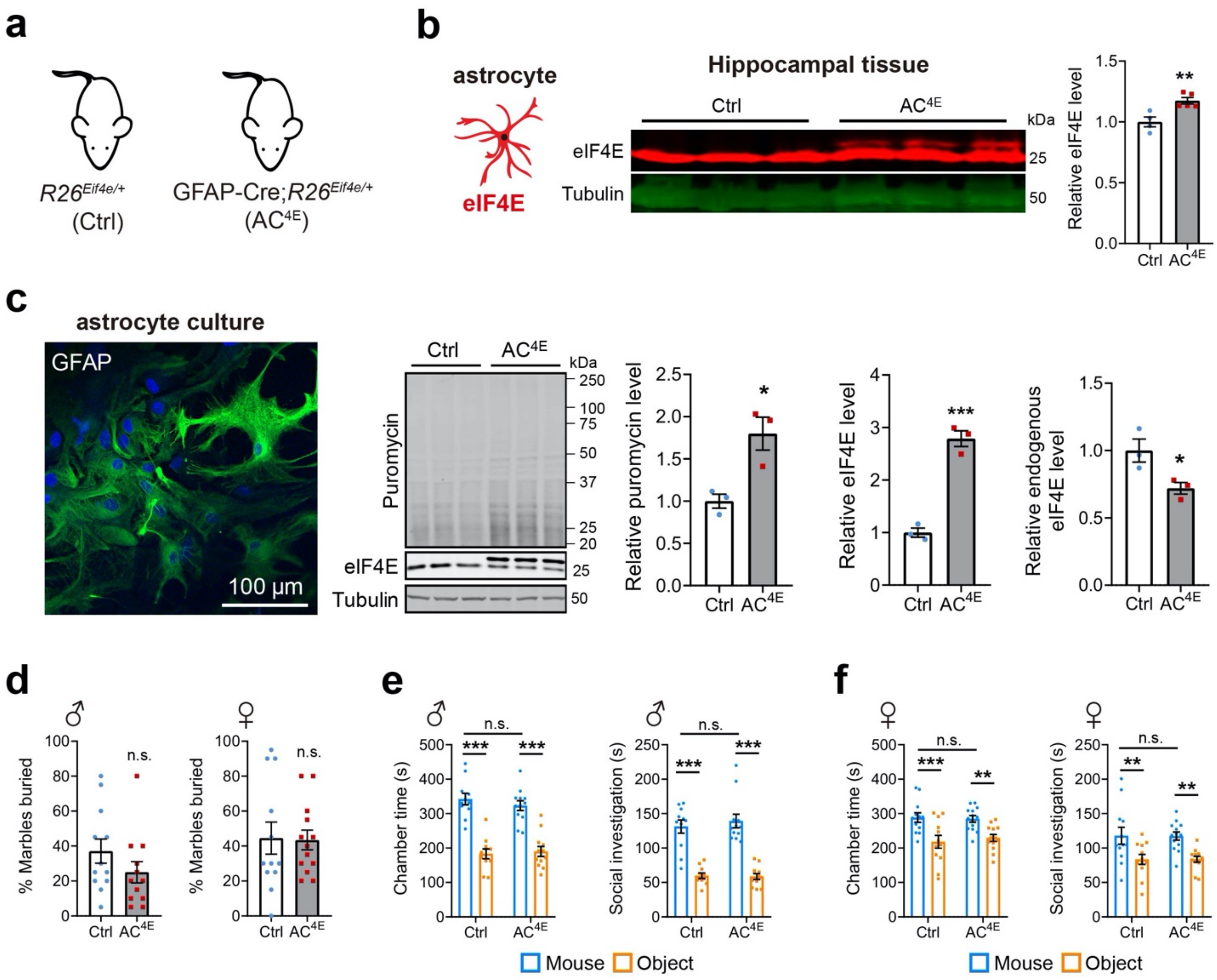

Supplementary Fig. 3. Normal sociability in mice overexpressing eIF4E in astrocytes. a, Overexpression of eIF4E in astrocytes using a GFAP-Cre mouse line. GFAP-Cre;*R26*$^{Eif4e/+}$ (AC^{4E}) mice overexpress eIF4E in astrocytes, whereas *R26*$^{Eif4e/+}$ mice serve as controls (Ctrl). **b**, Immunoblot and quantification of hippocampal eIF4E in control and AC^{4E} mice. Alpha tubulin was used as internal loading control. n = 4 for control and 5 for AC^{4E}. **p = 0.0069 by two-sided *t* test. **c**, Astrocytic eIF4E overexpression (***p = 0.0005) and protein synthesis (*p = 0.0197), as measured using eIF4E and puromycin immunoblots of cultured astrocytes isolated from control and AC^{4E} mice. Note that overexpression of eIF4E-Myc decreased endogenous eIF4E levels (*p = 0.0434). n = 3 per condition. Two-sided *t* test. **d**, Percentage of marbles buried by male and female mice. Male: n = 12 control mice and 12 AC^{4E} mice; Female: n = 12 control mice and 13 AC^{4E} mice. n.s., not significant by two-sided *t* test. **e** & **f**, Normal social behavior in male (e) and female (f) AC^{4E} mice revealed by three-chamber sociability tests. Male: n = 12 control mice and 12 AC^{4E} mice; Female: n = 12 control mice and 13 AC^{4E} mice. Two-way ANOVA with Fisher's LSD post-hoc test: **p < 0.01; ***p < 0.001; n.s., not significant. All data are shown as mean ± s.e.m. Source data are provided as a Source Data file.

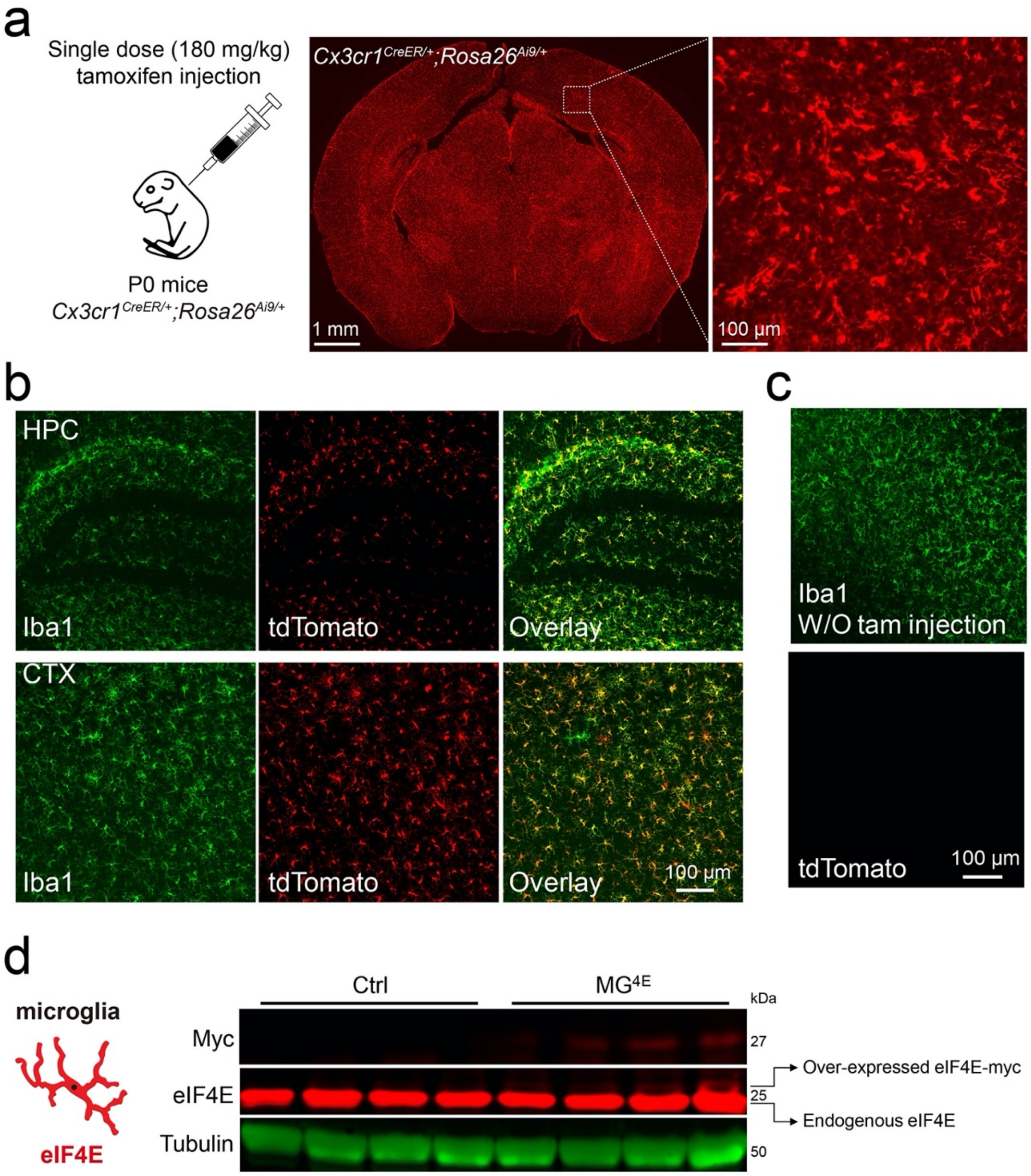

Supplementary Fig. 4. Transgenic eIF4E expression in microglia. a, Tamoxifen-induced eIF4E overexpression in microglia. Tamoxifen was injected into newborn pups. Confocal images showed tdTomato expression in *Cx3cr1*$^{CreER/+}$*;Rosa26*$^{Ai9/+}$ mice at P14. **b**, Confocal images showing co-expression of tdTomato with Iba1 in the hippocampus (HPC) and cortex (CTX) of *Cx3cr1*$^{CreER/+}$*;Rosa26*$^{Ai9/+}$ mice at P14. Representative images are from n = 3 mice. **c,** No expression of tdTomato in cortex of *Cx3cr1*$^{CreER/+}$*;Rosa26*$^{Ai9/+}$ mice in the absence of tamoxifen injection. Representative images are from n = 2 mice. **d**, Myc and eIF4E immunoblots of hippocampal extracts prepared from control and MG^{4E} mice. Alpha tubulin was used as an internal loading control. n = 4 control mice and 4 MG^{4E} mice. Source data are provided as a Source Data file.

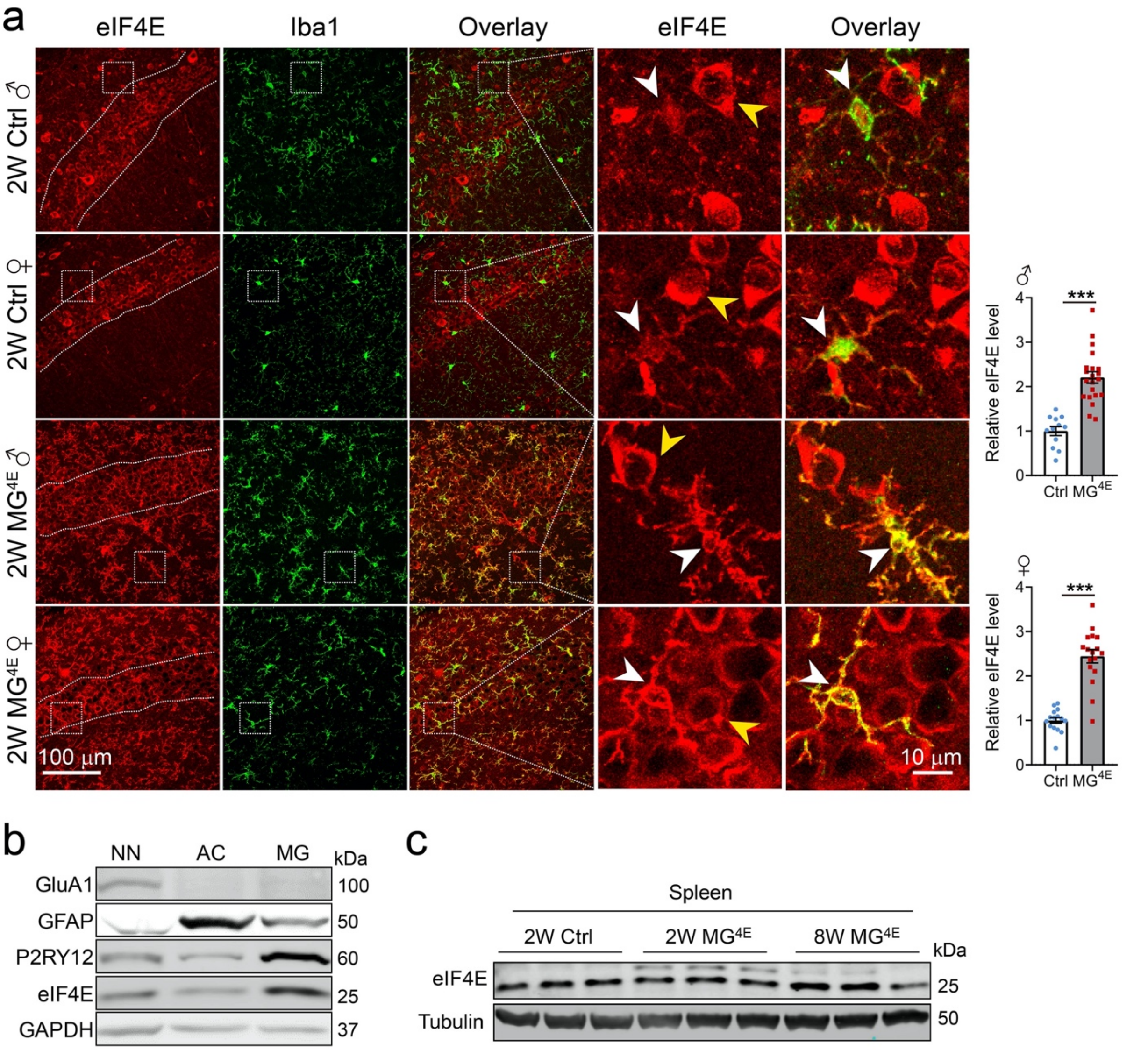

Supplementary Fig. 5. Overexpression of eIF4E in microglia. a, Increased eIF4E expression in hippocampal microglia of male and female MG^{4E} mice at P14, as revealed by eIF4E immunohistochemistry. The CA1 cell body layer in the left column of images is outlined by two dash lines. White arrowheads denote microglia and yellow arrowhead neurons. The dotted bar graphs show relative eIF4E immunoreactivity in microglia. Male, n = 12 microglia for control and n = 21 microglia for MG^{4E}; female, n = 15 microglia for control and n = 17 microglia for MG^{4E}. ***p < 0.001 by two-sided *t* test. **b**, Expression of endogenous eIF4E in primary neuronal (NN), astrocytic (AC) and microglial (MG) cultures. GluA1, GFAP and P2Y12 serve as markers for neurons, astrocytes and microglia, respectively. Cells were from three independent cultures. **c**, Expression of eIF4E and eIF4E-Myc in spleen extracts prepared from 2-week-old (2W) and 8-week-old (8W) MG^{4E} mice. n = 3 mice per group. All data are shown as mean ± s.e.m. Source data are provided as a Source Data file.

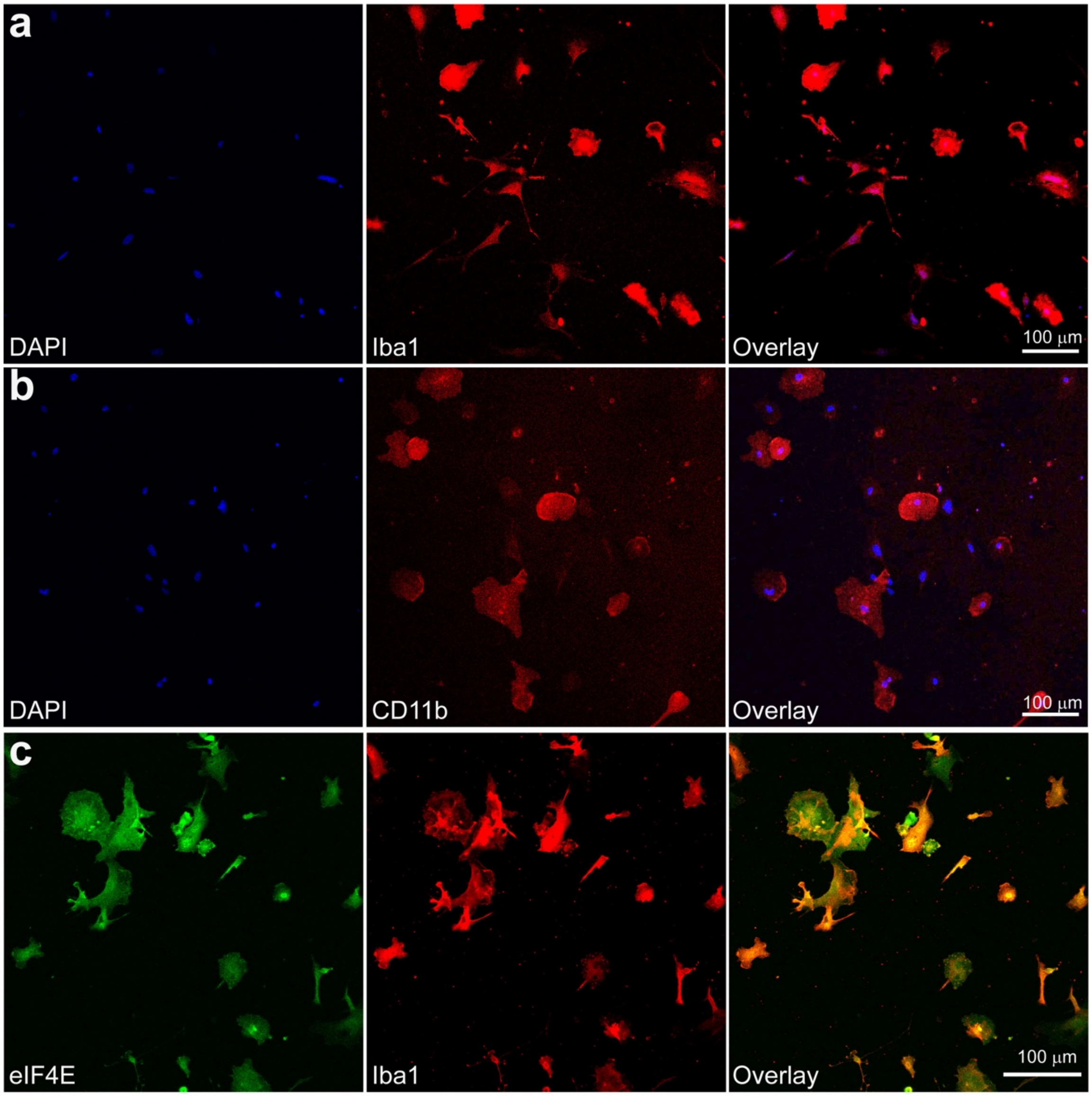

Supplementary Fig. 6. Culture of microglia purified with immunopanning. a, The majority of cultured cells were positive for microglia marker Iba1. **b**, Approximately 50% of cultured cells are positive for CD11b immunoreactivity. **c**, Endogenous eIF4E expression in cultured microglia, revealed by Iba1 and eIF4E double immunocytochemistry. Representative images are from four independent cultures.

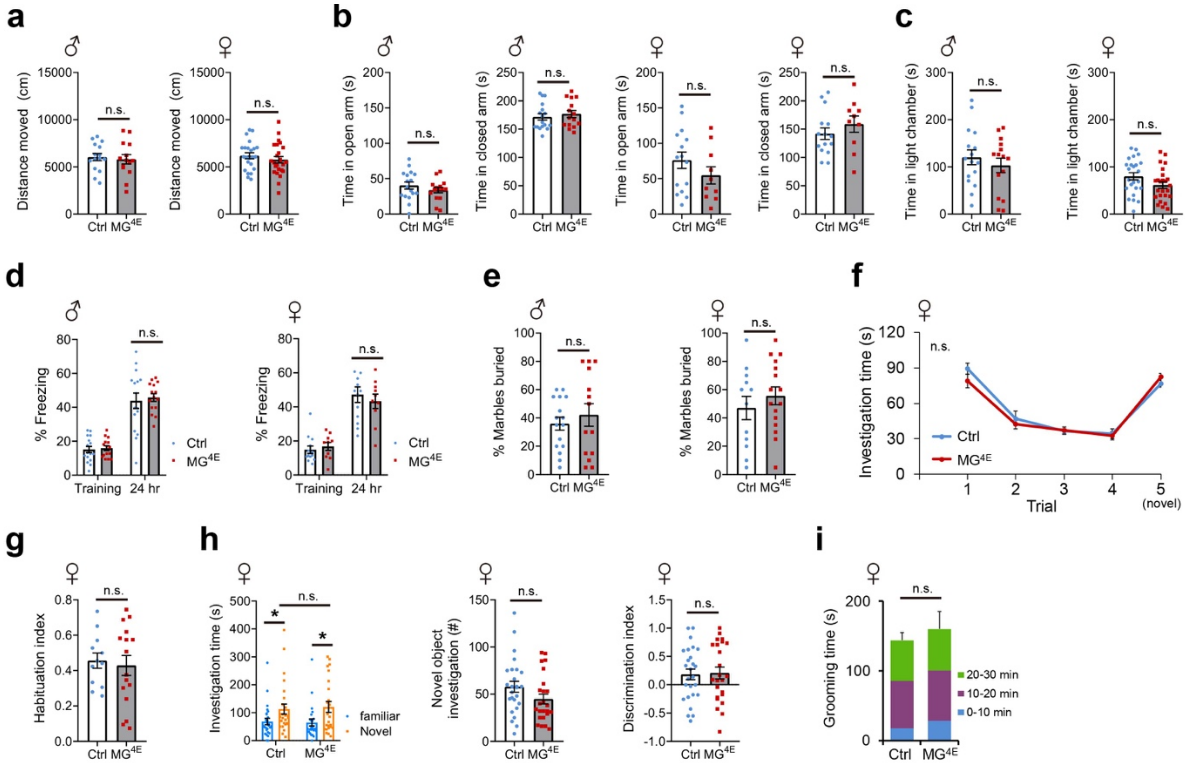

Supplementary Fig. 7. Behavioral analysis of MG^{4E} mice. a, Open field test. Male: n = 15 control mice and 14 MG^{4E} mice; Female: n = 23 control mice and 25 MG^{4E} mice. n.s., not significant by two-sided *t* test. **b**, Elevated plus maze test. Male: n = 17 control mice and 15 MG^{4E} mice; Female: n = 15 control mice and 10 MG^{4E} mice. n.s., not significant by two-sided *t* test. **c**, Time spent in the light chamber in light-dark box tests. Male: n = 16 control mice and 15 MG^{4E} mice; Female: n = 24 control mice and 25 MG^{4E} mice. n.s., not significant by two-sided *t* test. **d**, Normal contextual fear memory in male and female MG^{4E} mice. Male: n = 16 control mice and 15 MG^{4E} mice; Female: n = 13 control mice and 10 MG^{4E} mice. n.s., not significant by two-sided *t* test. **e**, Percentage of marbles buried. Male: n = 16 control mice and 14 MG^{4E} mice; Female: n = 12 control mice and 16 MG^{4E} mice. n.s., not significant by two-sided *t* test. **f**, Social habituation test in female control and MG^{4E} mice. n = 12 control mice and 16 MG^{4E} mice. Two-way ANOVA with Fisher's LSD post-hoc test, n.s., not significant. **g**, Habituation index. n.s., not significant by two-sided *t* test. **h**, Novel object recognition. n = 26 control mice and 24 MG^{4E} mice. Investigation time was analyzed by Two-way ANOVA with Fisher's LSD post-hoc test: *p = 0.0443 for Ctrl, *p = 0.0161 for MG^{4E} and n.s. = not significant. Investigation number and discrimination index were analyzed by two-sided *t* test. **i**, Self-grooming. The 30-min self-grooming test was divided into three 10-min segments. n = 10 control mice and 9 MG^{4E} mice. Two-sided *t* test was used for the whole 30-min test period, n.s., not significant. Two-way ANOVA (genotype x time, $F_{(2,51)}$ = 0.137, p = 0.872) with Fisher's LSD post-hoc test was used for each 10-min segment. All data are shown as mean ± s.e.m. Source data are provided as a Source Data file.

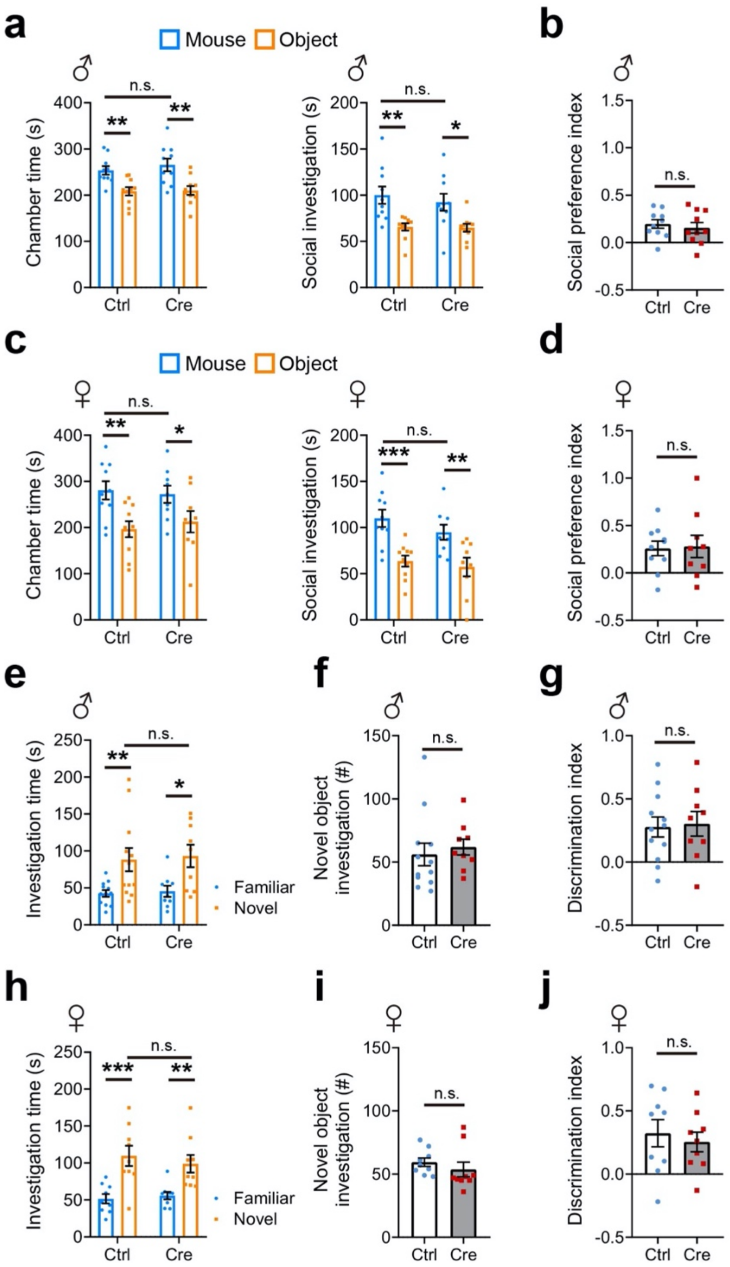

Supplementary Fig. 8. Normal sociability and object recognition in *Cx3cr1*$^{CreER/+}$ mice. Tamoxifen was injected into newborn pups of wild-type (Ctrl) and *Cx3cr1*$^{CreER/+}$ (Cre) mice, and sociability (**a-d**) and novel object recognition (**e-j**) tests were performed in 8-week-old male and female mice. Male: n = 10 control mice and 10 Cre mice; Female: n = 10 control mice and 9 Cre mice. Two-way ANOVA with Fisher's LSD post-hoc test was used for male chamber time (Ctrl, **p = 0.0047; Cre, **p = 0.0010), male social investigation (**p = 0.0017, *p = 0.010), female chamber time (**p = 0.0039, *p = 0.0465), female social investigation (***p = 0.0003, **p = 0.0042), male investigation time on objects (**p = 0.0062, *p = 0.01245), and female investigation time on objects (***p = 0.0002, **p = 0.0045). Two-sided *t* test was used in b (p = 0.5743), d (p = 0.8794), f (p = 0.6248), g (p = 0.8415), i (p = 0.4011) and j (p = 0.6063). n.s., not significant. All data are shown as mean ± s.e.m. Source data are provided as a Source Data file.

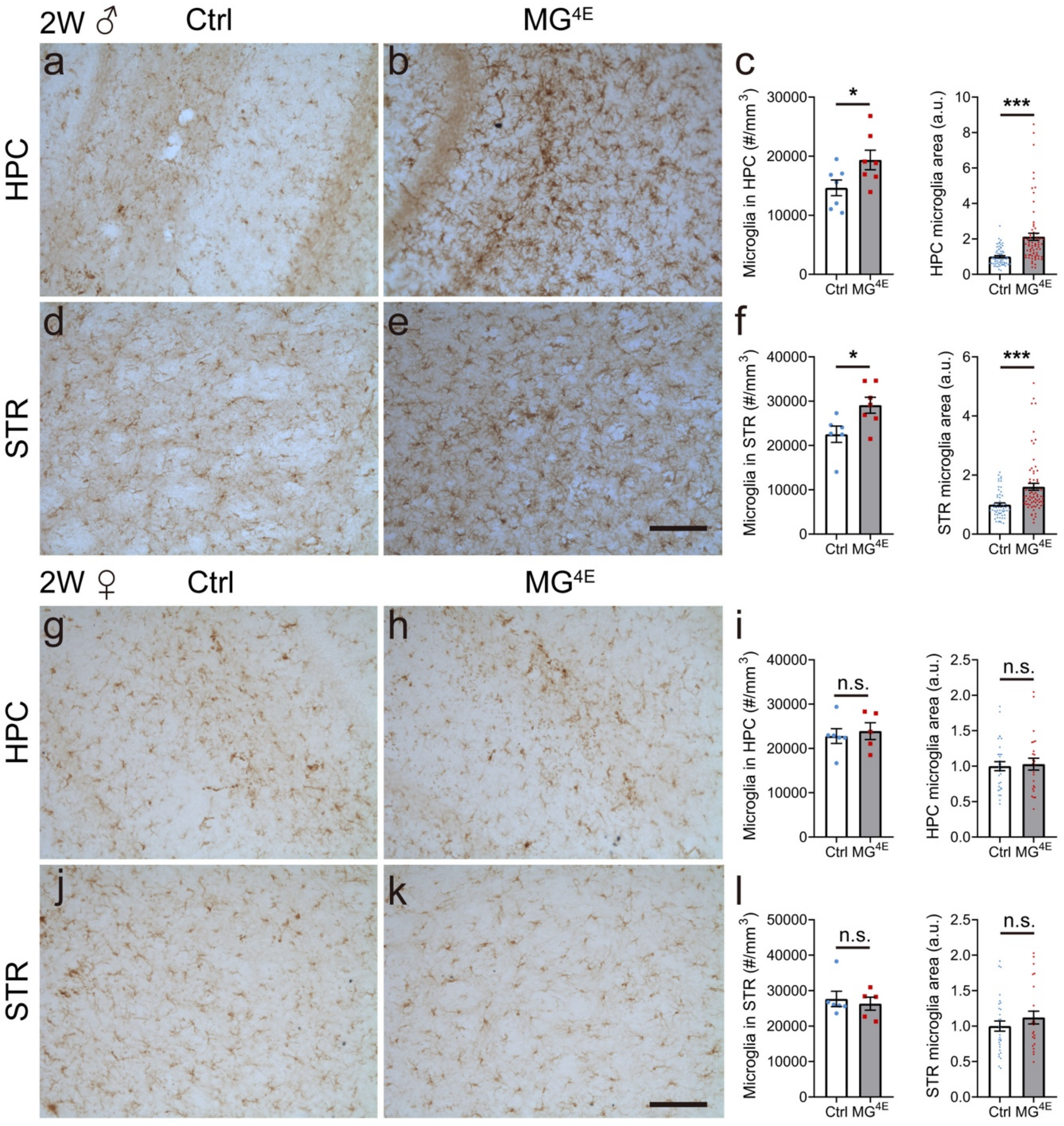

Supplementary Fig. 9. Increased protein synthesis changed microglial density and morphology only in male MG^{4E} mice. a-f, Increased microglia density and size in the hippocampus (HPC, a-c) and striatum (STR, d-f) of 2-week-old male MG^{4E} mice. 7 mice per genotype. Ten microglia in each brain region of each mouse were randomly selected for measurement of cell size (cross section area). Two-sided *t* test (c, *p = 0.0458, ***p < 0.001; f, *p = 0.0273, ***p < 0.001). Scale bar, 100 μm. **g-l**, Comparable microglial density and size in the hippocampus (g-i) and striatum (j-l) between female control and MG^{4E} mice. n = 6 control mice and 5 MG^{4E} mice. 5-10 microglia in each brain region of each mouse were randomly selected for measurement of cell size (cross section area). n.s., not significant by two-sided *t* test. Scale bars, 100 μm. All data are shown as mean ± s.e.m. Source data are provided as a Source Data file.

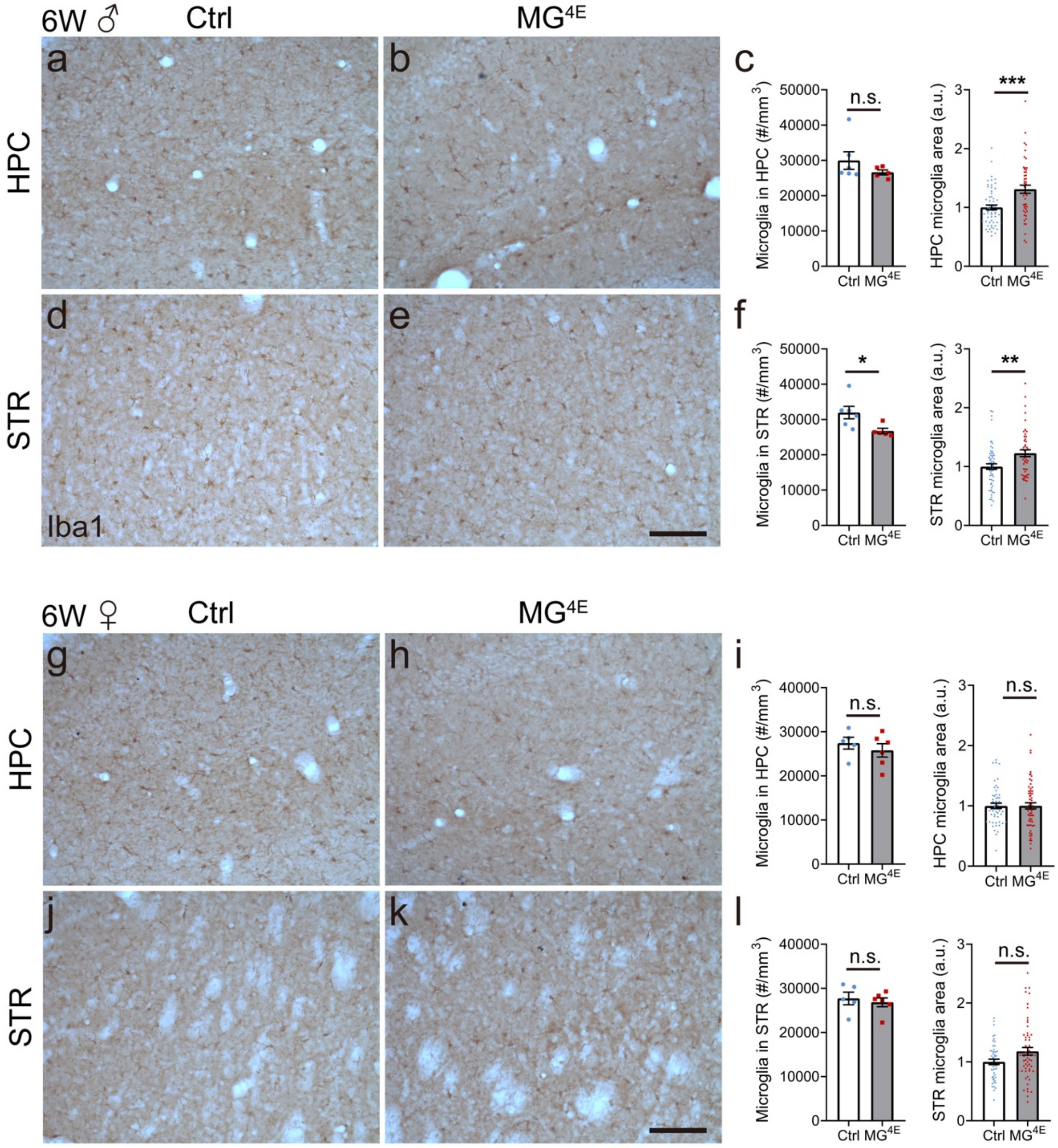

Supplementary Fig. 10. Microglial density and morphology in 6-week-old male and female MG^{4E} mice. a-f, Microglia density and size in the hippocampus (HPC; a-c) and striatum (STR; d-f) of 6-week-old male MG^{4E} mice. Ten microglia in each brain region of each mouse were randomly selected for measurement of cell size (cross section area) (6 mice per genotype). Two-sided *t* test: *p = 0.0322, **p = 0.0025, ***p = 0.0001, and n.s., not significant. Scale bar, 100 μm. **g-l,** Microglia density and size in the hippocampus (HPC; g-i) and striatum (STR; j-l) of 6-week-old female MG^{4E} mice. Ten microglia in each brain region of each mouse were randomly selected for measurement of cell size (5 control mice and 6 MG^{4E} mice). Two-sided *t*-test: n.s., not significant. Scale bars, 100 μm. All data are shown as mean ± s.e.m. Source data are provided as a Source Data file.

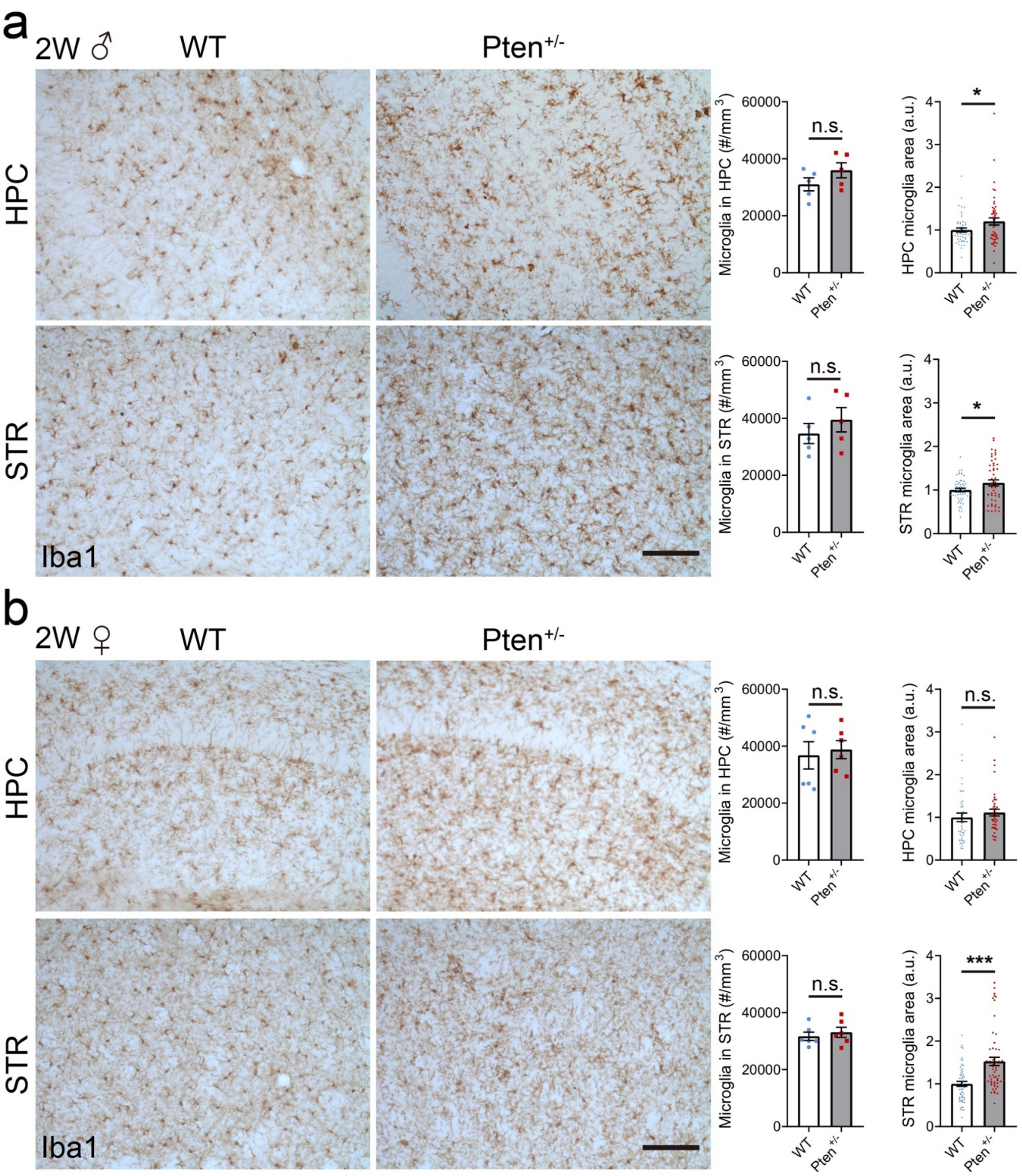

Supplementary Fig. 11. Altered microglial density and size in *Pten*$^{+/-}$ mice. Quantification of density and size of Iba1^{+} microglia in the hippocampus and striatum of 2-week-old male (a) and female (b) WT and *Pten*$^{+/-}$ mice. Male, n = 5 mice per genotype; female, n = 6 mice per genotype. 5-10 microglia in each brain region of each mouse were randomly selected for measurement of cell size (cross section area). Two-sided *t* test: a, *p = 0.0388 for HPC microglia area and *p = 0.0444 for STR microglia area; b, ***p < 0.001 and n.s., not significant. Scale bars, 100 μm. All data are shown as mean ± s.e.m. Source data are provided as a Source Data file.

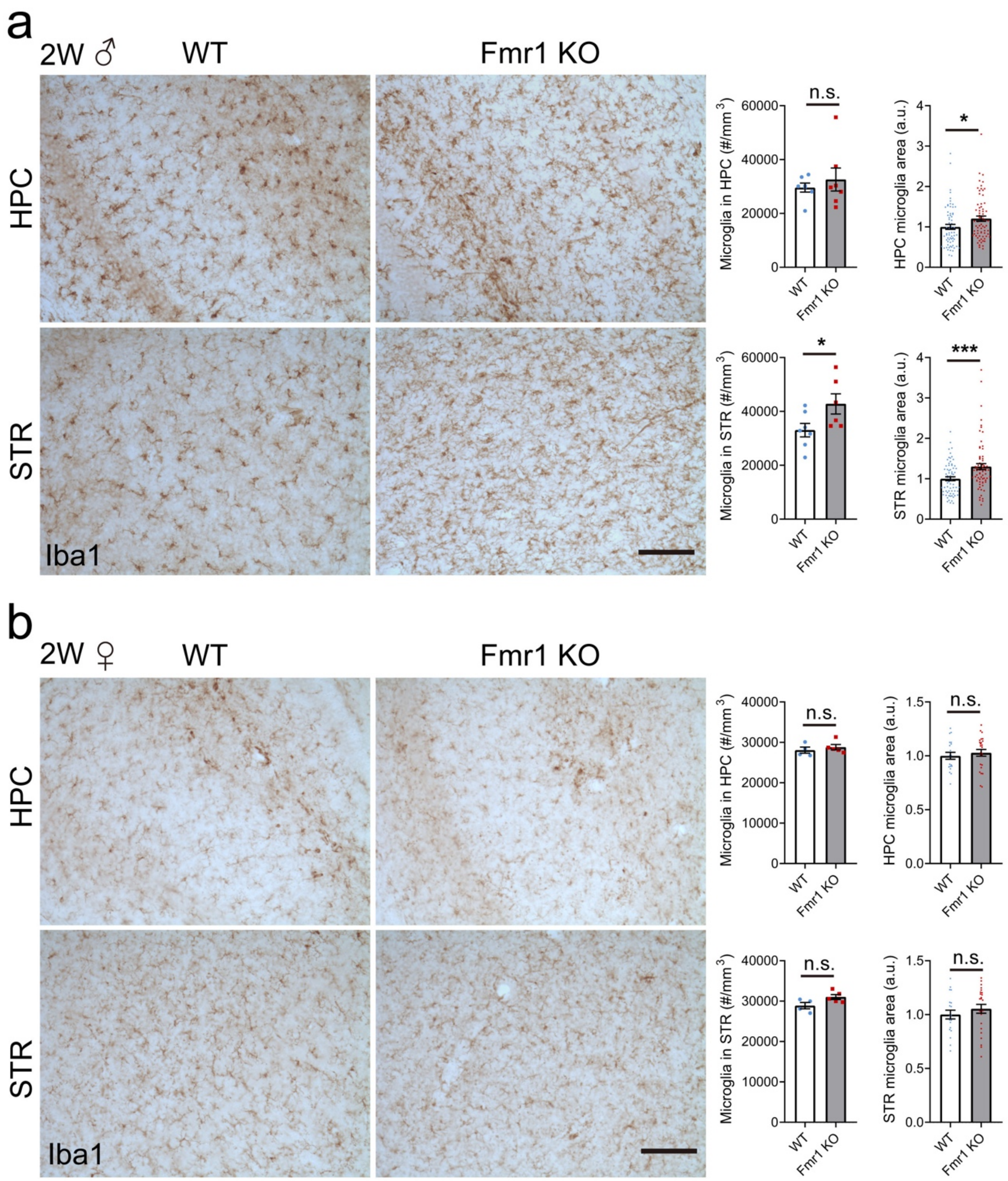

Supplementary Fig. 12. Altered microglial density and size in *Fmr1* KO mice. Quantification of density and size of Iba1^{+} microglia in the hippocampus and striatum of 2-week-old male (a) and female (b) WT and *Fmr1* KO mice. Male, n = 7 mice per genotype; Female, n = 4 WT mice and 5 *Fmr1* KO mice. 5-10 microglia in each brain region of each mouse were randomly selected for measurement of cell size (cross section area). Two-sided *t* test: male, HPC, p = 0.5216 for microglial density and *p = 0.0239 for microglial area; STR, *p = 0.0494 for microglial density and ***p = 0.0008 for microglial area; n.s., not significant. Scale bars, 100 μm. All data are shown as mean ± s.e.m. Source data are provided as a Source Data file.

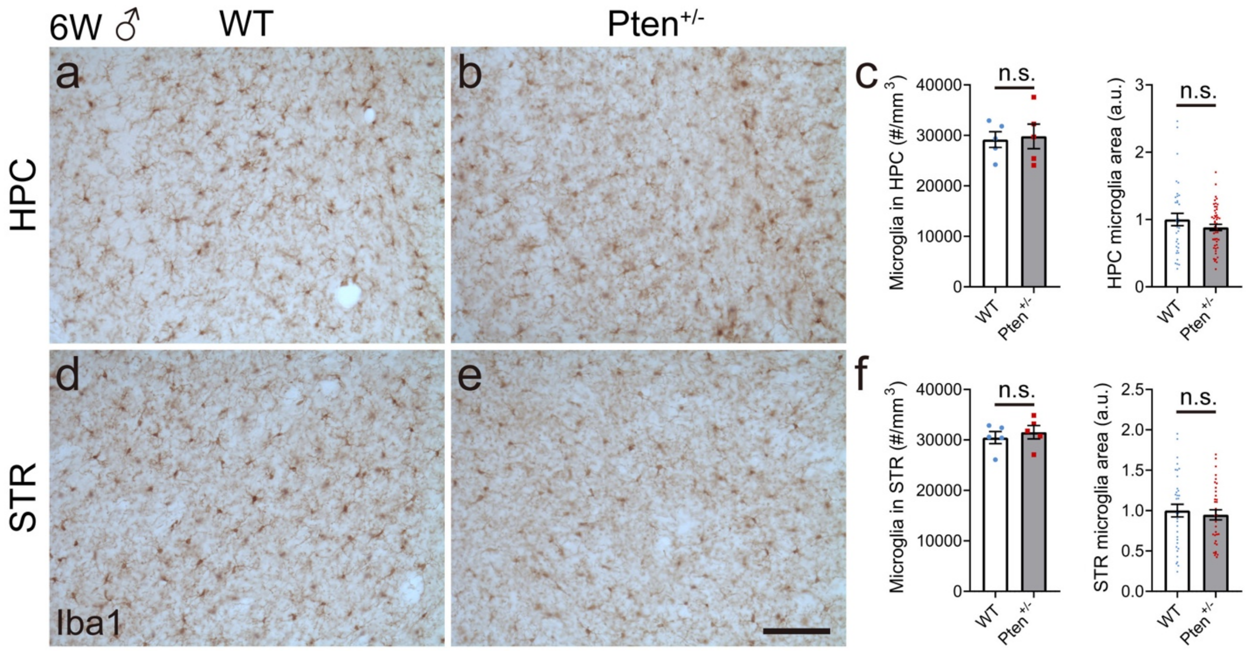

Supplementary Fig. 13. Microglial density and morphology in 6-week-old male WT and *Pten*$^{+/-}$ mice. Quantification of density and size of Iba1^{+} microglia in the hippocampus (a-c) and striatum (d-f) of 6-week-old male WT and *Pten*$^{+/-}$ mice. n = 5 mice per genotype. 5-10 microglia in each brain region of each mouse were randomly selected for measurement of cell size (cross section area). Two-sided *t* test: n.s., not significant. Scale bar, 100 µm. All data are shown as mean ± s.e.m. Source data are provided as a Source Data file.

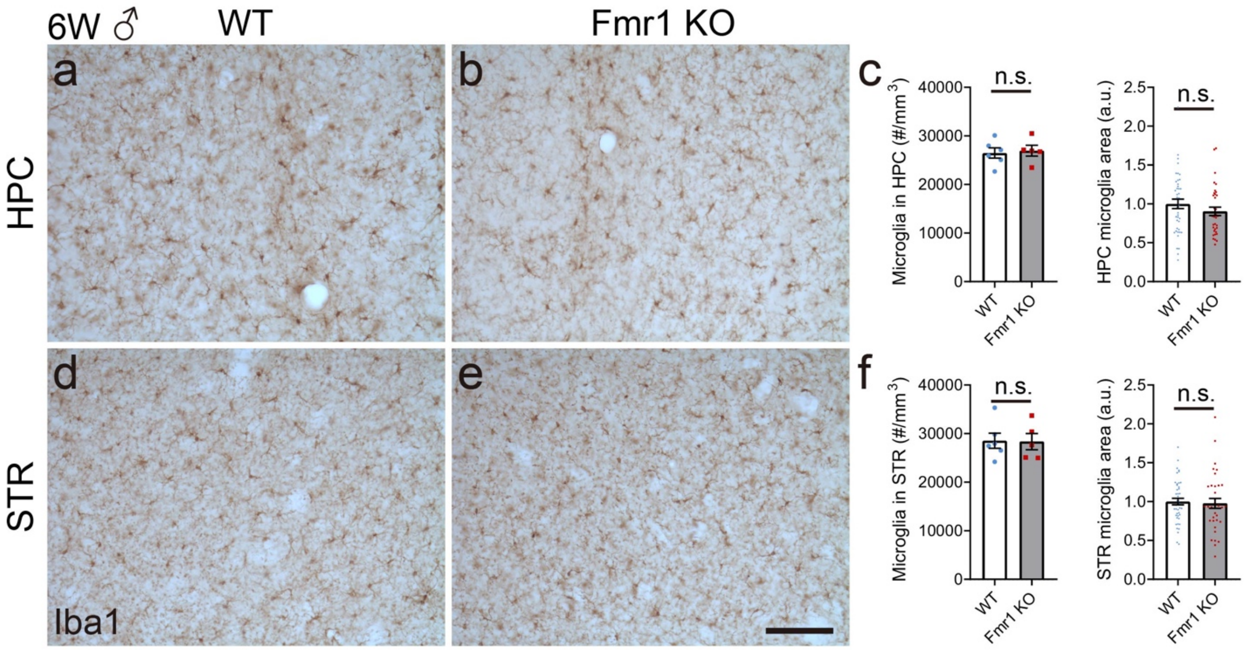

Supplementary Fig. 14. Microglial density and morphology in 6-week-old male WT and *Fmr1* KO mice. Quantification of density and size of Iba1$^+$ microglia in the hippocampus (a-c) and striatum (d-f) of 6-week-old male WT and *Fmr1* KO mice. n = 6 WT mice and 5 *Fmr1* KO mice. 5-10 microglia in each brain region of each mouse were randomly selected for measurement of cell size (cross section area). Two-sided *t* test: n.s., not significant. Scale bar, 100 μm. All data are shown as mean ± s.e.m. Source data are provided as a Source Data file.

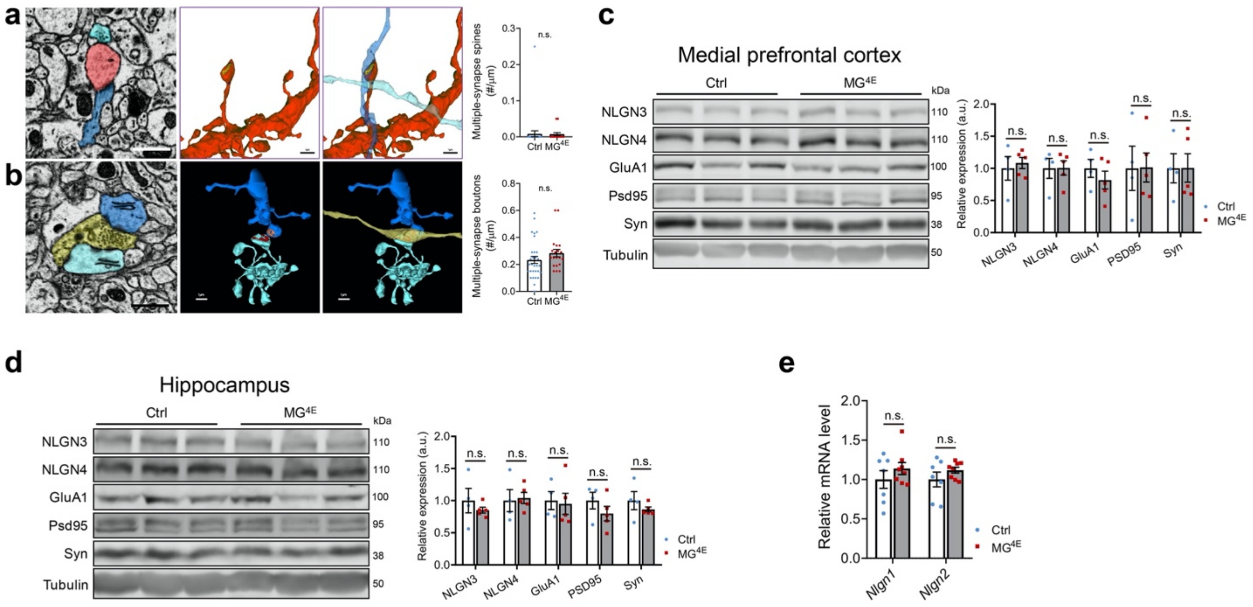

Supplementary Fig. 15. Synaptic multiplicity and expression of synaptic proteins in male MG^{4E} mice. a, Example and quantification of multiple synapses formed on a single spine in the mPFC of 6-week-old male control and MG^{4E} mice. n = 5 control mice and 6 MG^{4E} mice. n.s., not significant by two-sided *t* test. **b**, Example and quantification of multiple synapses formed on a single bouton in the mPFC of 6-week-old male control and MG^{4E} mice. n = 5 control mice and 6 MG^{4E} mice. n.s., not significant by two-sided *t* test. **c**, Immunoblots and quantification of synaptic proteins in the mPFC of 6-week-old male control and MG^{4E} mice. n = 4 control mice and 5 MG^{4E} mice. n.s., not significant by two-sided *t* test. **d**, Immunoblots and quantification of synaptic proteins in the hippocampus of 6-week-old male control and MG^{4E} mice. n = 4 control mice and 5 MG^{4E} mice. n.s., not significant by two-sided *t* test. **e**, Levels of *Nlgn1* and *Nlgn2* mRNA in the hippocampus of 6-week-old male control and MG^{4E} mice. n = 7 control mice and 8 MG^{4E} mice. n.s., not significant by two-sided *t* test. All data are shown as mean ± s.e.m. Source data are provided as a Source Data file.

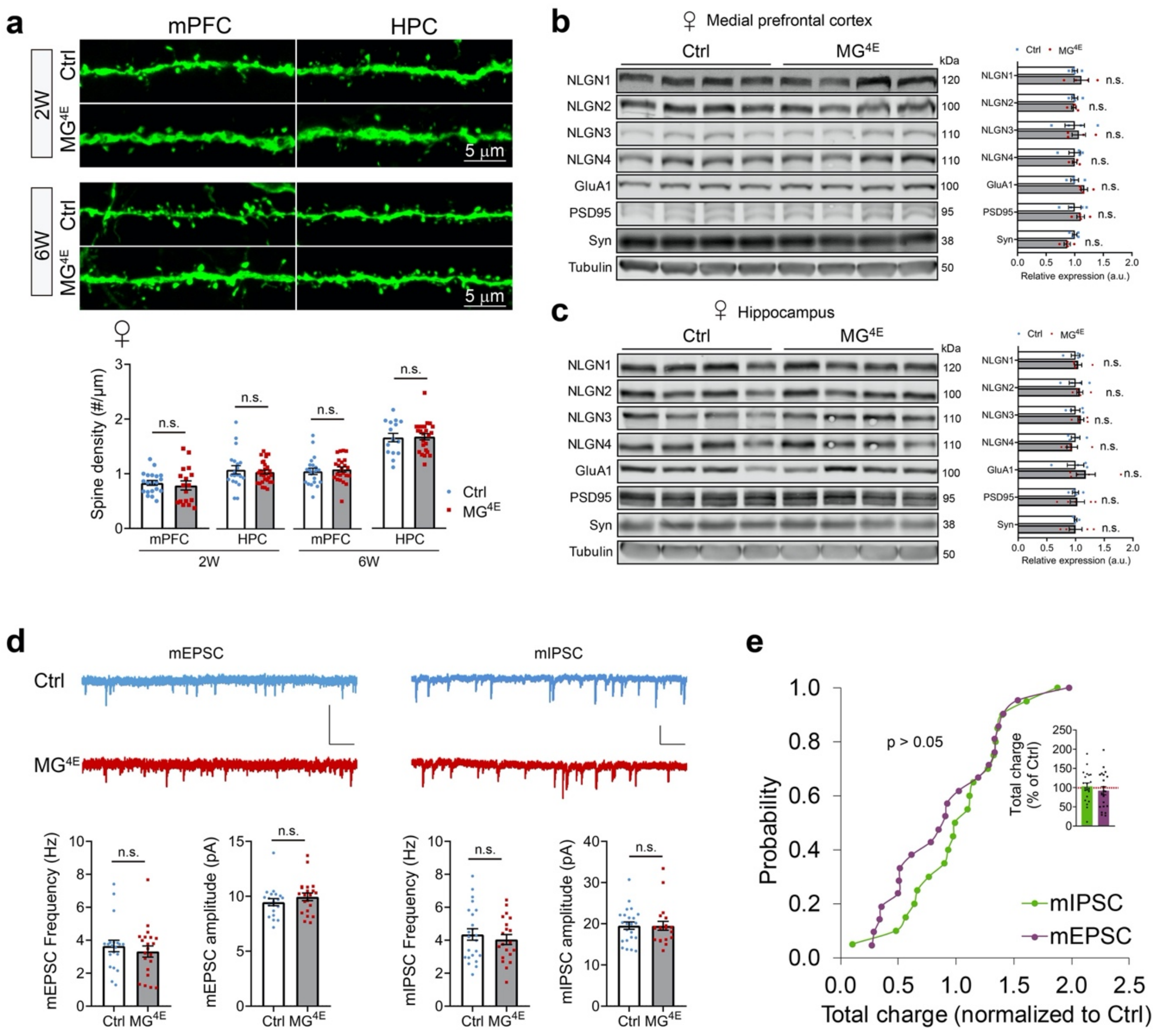

Supplementary Fig. 16. Spine density and synaptic function in female MG^{4E} mice. a, Spine density in mPFC layer 5 neurons and hippocampal CA1 neurons of 2-week-old (2W) and 6-week-old (6W) female control and MG^{4E} mice. 2W, 4 control mice and 6 MG^{4E} mice; 6W, 4 control mice and 6 MG^{4E} mice; 2-5 neurons in each brain region per mouse. n.s., not significant by two-sided *t* test. Scale bars, 5 µm. **b** & **c**, Immunoblots and quantification of synaptic proteins in the mPFC and hippocampus of 6-week-old female control and MG^{4E} mice. n = 4 control mice and 5 MG^{4E} mice. n.s., not significant by two-sided *t* test. **d**, mEPSCs and mIPSCs recorded in mPFC layer 5 neurons of female control and MG^{4E} mice at 6-7 weeks of age. For mEPSCs, n = 20 cells from 6 control mice and 21 cells from 5 MG^{4E} mice, scale bars, 50 pA (vertical) and 0.5 s (horizontal); for mIPSCs, n = 23 cells from 6 control mice and 20 cells from 4 MG^{4E} mice, scale bars, 25 pA (vertical) and 0.5 s (horizontal). n.s., not significant by two-sided *t* test. **e**, Relative changes in mEPSC and mIPSC total charge transfer in female MG^{4E} mice, normalized to control mice. p = 0.6325 by two-sided Kolmogorov-Smirnov test. All data are shown as mean ± s.e.m. Source data are provided as a Source Data file.

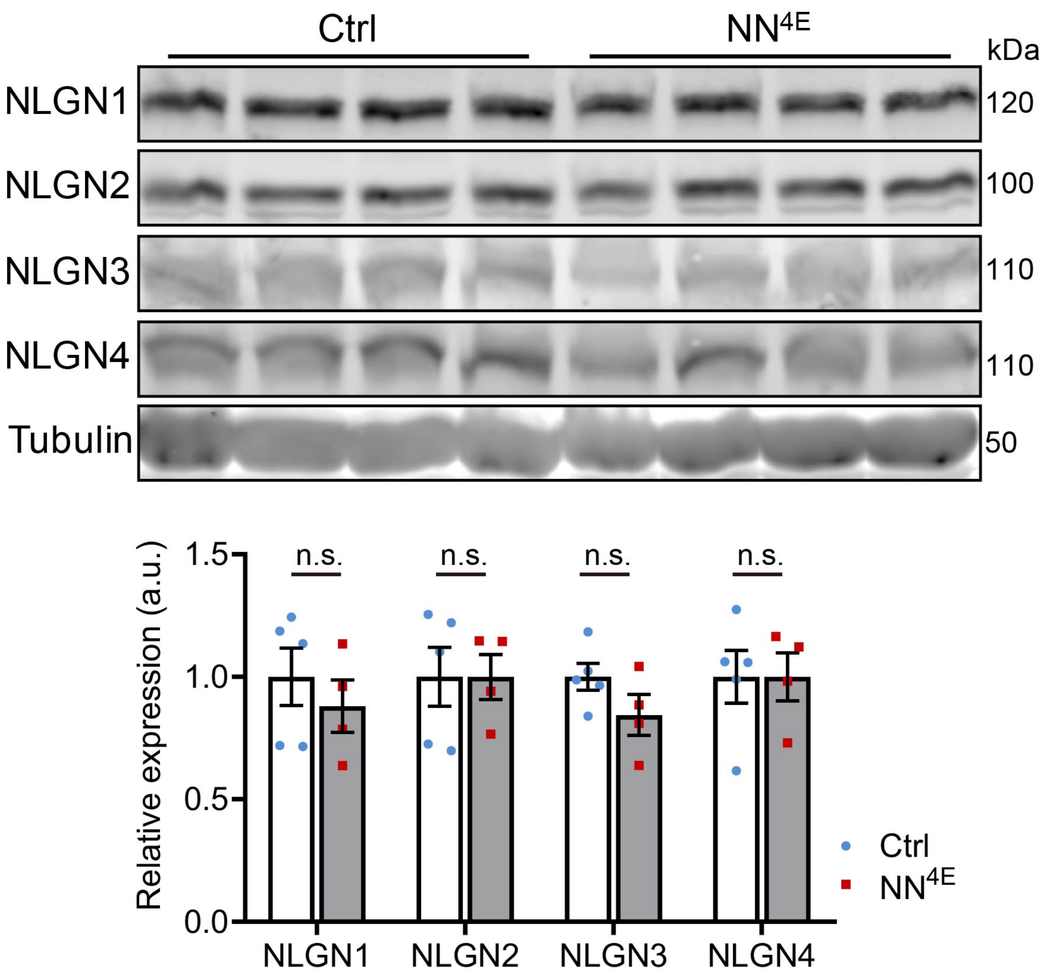

Supplementary Fig. 17. Levels of neuroligins in NN^{4E} mice. Immunoblots and quantification of neuroligin 1-4 in the hippocampus of 6-week-old male control and NN^{4E} mice. n = 5 control mice and 4 NN^{4E} mice. n.s., not significant by two-sided *t* test. All data are shown as mean ± s.e.m. Source data are provided as a Source Data file.

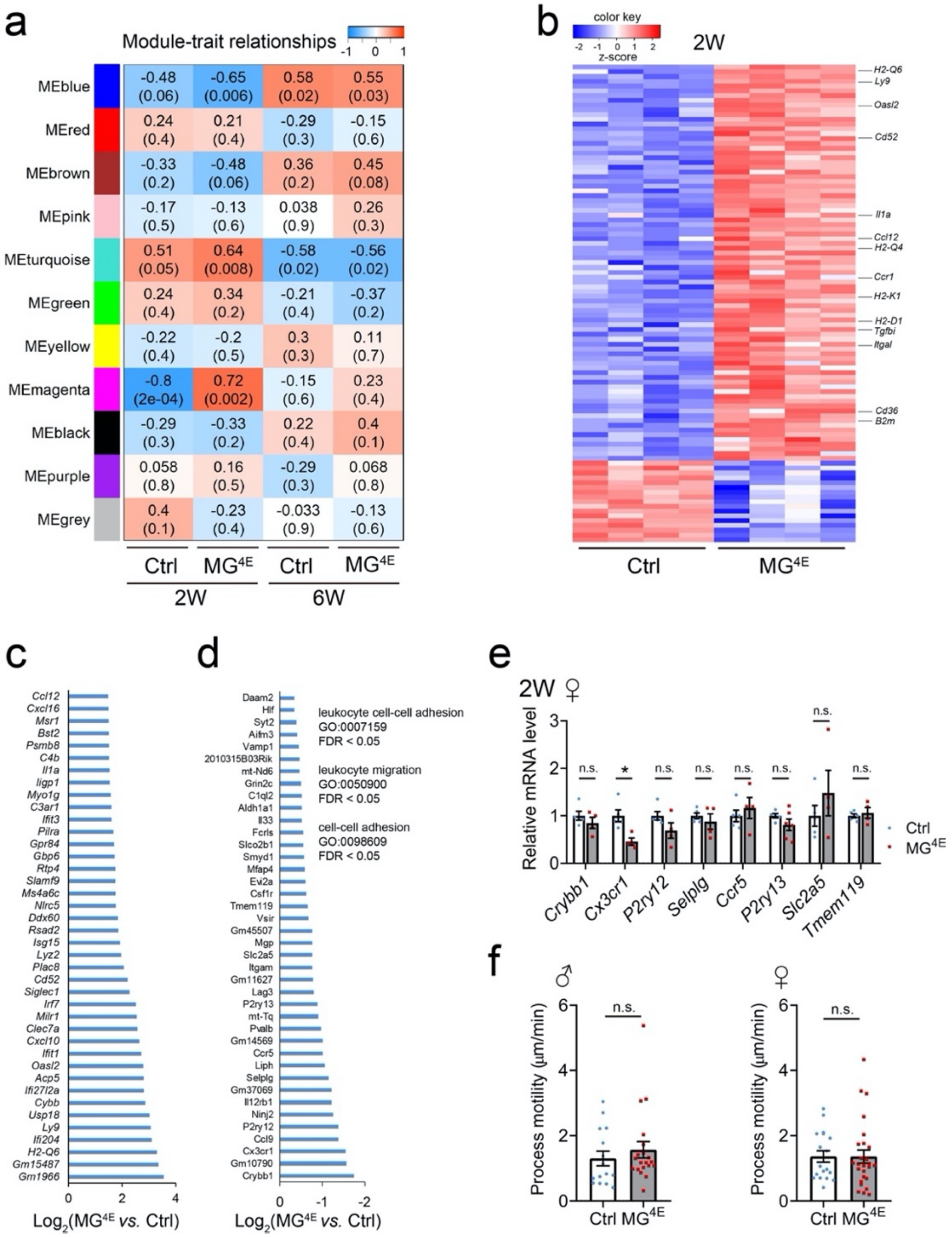

Supplementary Fig. 18. Altered gene expression profile in MG^{4E} mice. **a**, Module-trait relationships revealed by WGCNA analysis. **b**, Heatmaps of top100 differentially expressed genes (DEGs) in 2-week-old male control and MG^{4E} mice (FDR < 0.05). **c**, List of top 40 up-regulated DEGs in 2-week-old male MG^{4E} mice (FDR < 0.05). **d**, List of top 40 down-regulated DEGs and its top significantly enriched terms for biological processes (FDR < 0.05). **e**, Levels of mRNAs for selected genes in 2-week-old female control and MG^{4E} mice. Levels of *Cx3cr1* mRNA were lower in MG^{4E} mice due to disruption of one *Cx3cr1* allele. n = 5 control mice and 4 MG^{4E} mice. *p = 0.0101, and n.s., not significant by two-sided *t* test. **f**, Motility of male and female microglial process under physiological conditions (pre-ATP treatment). For male, n = 15 microglia from 3 control mice and 20 microglia from 3 MG^{4E} mice; for female, n = 18 microglia from 3 control mice and 26 microglia from 3 MG^{4E} mice. n.s., not significant by two-sided *t* test. Data in e and f are shown as mean ± s.e.m. Source data are provided as a Source Data file.

Supplementary Table 1. List of PCR primers

Gene	Accession number	Forward sequence	Reverse sequence
Cx3cr1	NM_009987.4	CAGCATCGACCGGTACCTT	GCTGCACTGTCCGGTTGTT
Csf1r	NM_001037859.2	TAAGCAAGATCTGGACAAAGA	CCGCTGGTCAACAGCACGTTT
P2ry12	NM_027571.3	TGAAGACCACCAGGCCATTT	AGGCCCAGATGACAACAGAAA
P2ry13	NM_028808.3	TGGGTTGAGCTAGTAACTGCC	TTGTCCCGAGCATCAGCTTT
Selplg	NM_009151.3	AAGTGTCTGGCAGTGTGGAC	ATGGTACCGTGCCCAACAG
Tmem119	NM_146162.2	GTGTCTAACAGGCCCCAGAA	AGCCACGTGGTATCAAGGAG
CCR5	NM_009917.5	GCTGCCTAAACCCTGTCATC	GTTCTCCTGTGGATCGGGTA
Clec7a	NM_001309637.1	CCCAACTCGTTTCAAGTCAG	AGACCTCTGATCCATGAATCC
Cd52	NM_013706.2	GGTTGTGATTCAGATACAAACAG	GAGGTAGAAGAGGCACATTAAG
crybb1	NM_023695.3	TCCCAGGAACATAAGATCTGC	ACGGTCACAGAAGCCATAAAC
Slc2a5	NM_019741.3	TCTCTTCCAACGTGGTCCCTA	GAGACTCCGAAGGCCAAACAG
Slco2b1	NM_175316.3	TTGGCATCGGTGGTGTGCCC	ATGCCTCCTTCTGGCATCCGGT
Fcrls	NM_030707.3	GTCGCTGGGGCACTGTATGT	GCACAGGCAGAGCTTCATCAA
Il1A	NM_010554.4	CTGATGAAGCTCGTCAGGCAG	TGGTGCTGAGATAGTGTTTGTC
Ccl2	NM_011333.3	TGAAGTTGACCCGTAAATCTGAA	AGGCATCACAGTCCGAGTC
Nlgn1	NM_001163387.2	ACAGGAGAACATCGTTTCCAGCCT	ATACAGGAGCAAACTGAGTGGCGT
Nlgn2	NM_001364137.1	ACTATCTTTGGGTCTGGTGC	ATGAGCATGTCGTAGTTGAGG
LyZ	NM_017372.3	TGGGATCAATTGCAGTGCT	CACCACCCTCTTTGCACATT
Actin	NM_007393.5	TGTGATGGTGGGAATGGGTCAGAA	TGTGGTGCCAGATCTTCTCCATGT
