# Supplementary material for: Elevated protein synthesis in microglia causes autism-like synaptic and behavioral aberrations
Source: Nat Commun. 2020 Apr 14;11:1797. doi: 10.1038/s41467-020-15530-3 (PMC7156673; doi:10.1038/s41467-020-15530-3)
Supplement: Supplementary file 3 — Reporting Summary [file 41467_2020_15530_MOESM3_ESM.pdf]

nature research

Corresponding author(s): Baoji Xu

Last updated by author(s): Feb 25, 2020

Reporting Summary

Nature Research wishes to improve the reproducibility of the work that we publish. This form provides structure for consistency and transparency in reporting. For further information on Nature Research policies, see Authors & Referees and the Editorial Policy Checklist .

Statistics

For all statistical analyses, confirm that the following items are present in the figure legend, table legend, main text, or Methods section.

n/a	Confirmed	
☐	☒	The exact sample size (*n*) for each experimental group/condition, given as a discrete number and unit of measurement
☐	☒	A statement on whether measurements were taken from distinct samples or whether the same sample was measured repeatedly
☐	☒	The statistical test(s) used AND whether they are one- or two-sided<br>*Only common tests should be described solely by name; describe more complex techniques in the Methods section.*
☐	☒	A description of all covariates tested
☐	☒	A description of any assumptions or corrections, such as tests of normality and adjustment for multiple comparisons
☐	☒	A full description of the statistical parameters including central tendency (e.g. means) or other basic estimates (e.g. regression coefficient) AND variation (e.g. standard deviation) or associated estimates of uncertainty (e.g. confidence intervals)
☐	☒	For null hypothesis testing, the test statistic (e.g. *F*, *t*, *r*) with confidence intervals, effect sizes, degrees of freedom and *P* value noted<br>*Give P values as exact values whenever suitable.*
☒	☐	For Bayesian analysis, information on the choice of priors and Markov chain Monte Carlo settings
☒	☐	For hierarchical and complex designs, identification of the appropriate level for tests and full reporting of outcomes
☒	☐	Estimates of effect sizes (e.g. Cohen's *d*, Pearson's *r*), indicating how they were calculated

Our web collection on statistics for biologists contains articles on many of the points above.

Software and code

Policy information about availability of computer code

Data collection	Image Studio Lite Ver 4.0 (Western blot); EthoVision XT (Behavior); NIS-Elements Confocal v4.6(Immunoflourescence); pCLAMP 10 (Electrophysiology)
Data analysis	Image Studio Lite Ver 4.0 (Western blot); Stereo Investigator 10 (cell counting), Amira 6.0 (3D microglial reconstruction and microglia engulfment); NIS-Elements Advanced Research v4.6(Immunoflourescence); EthoVision XT (Behavior); Image J version 1.52h and Fiji version 1.52i plugins TrakEM2 software (SB-SEM); RStudio Version 1.1.456 (RNA-seq); STAR version 2.5.2a aligner (RNA-seq); Bioconductor 3.10 packages edgeR (RNA-seq); DAVID 6.8 (RNA-seq); Generic GO Term Finder (RNA-seq); SPSS 19 (Statistics)

For manuscripts utilizing custom algorithms or software that are central to the research but not yet described in published literature, software must be made available to editors/reviewers. We strongly encourage code deposition in a community repository (e.g. GitHub). See the Nature Research guidelines for submitting code & software for further information.

Data

Policy information about availability of data

All manuscripts must include a data availability statement. This statement should provide the following information, where applicable:

- Accession codes, unique identifiers, or web links for publicly available datasets
- A list of figures that have associated raw data
- A description of any restrictions on data availability

RNA sequencing data have been deposited to the Sequence Read Archive (SRA) at NCBI and are available at the accession number PRJNA609402. The data that support the findings of this study are available from the corresponding author upon reasonable request. The source data for all figures are provided with the paper as a Source Data file.

Field-specific reporting

Please select the one below that is the best fit for your research. If you are not sure, read the appropriate sections before making your selection.

[x] Life sciences [ ] Behavioural & social sciences [ ] Ecological, evolutionary & environmental sciences

For a reference copy of the document with all sections, see nature.com/documents/nr-reporting-summary-flat.pdf

Life sciences study design

All studies must disclose on these points even when the disclosure is negative.

Sample size	No sample-size calculation was performed. The sample sizes were selected based on published studies (An et al. cell, 2008; Gkogkas et al. Nature 2013; Santini et al. Nature 2013)
Data exclusions	Exclusion criteria for experimental data points were pre-determined as follows. For electrophysiology, only neurons with Ra < 25 MΩ were recorded. For serial block-face scanning electron microscopy experiment, we avoided all dendritic segments with few or no spines (so that our analysis was restricted to pyramidal neurons). Outliers for behavioral testings were identified as being greater than two and a half standard deviations from the mean and excluded from statistical analysis. One mouse with stereotaxic injection of FAM-Aβ was excluded due to no Aβ aggregates were found in the hippocampus.
Replication	Multiple mice (n) were used for every experiment. All data in this study were collected from more than 3 independent experiments, and were reliably reproduced.
Randomization	For behavioral tests, mice were chosen based on genotypes. For microglial and neuronal morphology studies, cells from each mouse were randomly selected.
Blinding	Wherever possible (i.e. with the exception of Western Blotting experiments, where samples were grouped by genotypes), analyses were performed by blinded observers and/or software-automated analyses.

Reporting for specific materials, systems and methods

We require information from authors about some types of materials, experimental systems and methods used in many studies. Here, indicate whether each material, system or method listed is relevant to your study. If you are not sure if a list item applies to your research, read the appropriate section before selecting a response.

Materials & experimental systems

n/a	Involved in the study	
[ ]	[x]	Antibodies
[ ]	[x]	Eukaryotic cell lines
[x]	[ ]	Palaeontology
[ ]	[x]	Animals and other organisms
[x]	[ ]	Human research participants
[x]	[ ]	Clinical data

Methods

n/a	Involved in the study	
[x]	[ ]	ChIP-seq
[x]	[ ]	Flow cytometry
[x]	[ ]	MRI-based neuroimaging

Antibodies

Antibodies used	rabbit anti-Iba1 (Wako, 019-19741, 1:500); mouse anti-alpha-tubulin (Sigma-Aldrich T6074, 1:10000); mouse anti-beta-actin (Sigma-Aldrich A5441, 1:10000); mouse anti-PSD95 (Thermo Fisher Scientific MA1-045; 1:1000); rabbit anti-eIF4E (Cell Signaling Technology, #2067, 1:1000); rabbit anti-GluA1 (Millipore AB1504, 1:1000); rabbit anti-Myc (Cell Signaling Technology, #2278, 1:1000); rabbit anti-synaptophysin (Invitrogen, MA5-14532, 1:1000); mouse anti-neuroligin 1 (Synaptic Systems, 129111, 1:1000); rabbit anti-neuroligin 2 (Synaptic Systems, 129202, 1:1000); mouse anti-neuroligin 3 (Synaptic Systems, 129311, 1:1000); mouse anti-neuroligin 4 (Synaptic Systems, 129403, 1:1000); mouse anti-CD11b (Invitrogen, 12-0112-82, 1:100); goat anti-Iba1 (Abcam, ab5076, 1:500); rat anti-mouse CD45 antibody (Invitrogen, Cat. 17045182, 1:100); chicken anti-Homer1 (Synaptic Systems, 160006, 1:500); mouse anti-GFAP (MA5-12023, Thermo Fisher Scientific, 1:1000); rabbit anti-P2RY12 (702516, Thermo Fisher Scientific, 1:1000); rabbit anti-eIF4E antibody (A301-154A, Bethyl Laboratories, 1:100); mouse anti-eIF4E (sc-9976, Santa Cruz Biotechnology, 1:100); mouse anti-puromycin (1:2000, Millipore, MABE343); rabbit anti-eIF4G (Cell Signaling Technology, #2498, 1:1000); biotinylated goat anti-rabbit secondary antibody (1:200, Vector, BA-1000); Alexa Fluor 488-conjugated secondary antibody (711-545-152, Jackson ImmunoResearch, 1:500); Alexa Fluor 594-conjugated secondary antibody (703-585-155, Jackson ImmunoResearch, 1:500); Alexa Fluor 649-conjugated secondary antibody (711-605-152, Jackson ImmunoResearch, 1:500)
Validation	All antibodies are commercially available and selected according to the antibody validation which are available on the manufacturer's publicly accessible data sheets.

Eukaryotic cell lines

Policy information about cell lines

Cell line source(s)	HEK293 cells were from ATCC
Authentication	The cell line used was not authenticated
Mycoplasma contamination	Not tested
Commonly misidentified lines (See ICLAC register)	No commonly misidentified cell lines were used in the study.

Animals and other organisms

Policy information about studies involving animals; ARRIVE guidelines recommended for reporting animal research

Laboratory animals	Many mouse strains used in this study were obtained from the Jackson Laboratory: Syn1-Cre (Stock No: 003966), Gfap-Cre (Stock No: 024098), Thy1-GFP (Stock No: 007788), Rosa26Ai9/+ (Stock No: 007909), Fmr1 knockout (Stock No: 003025), CD68-EGFP (Stock No: 026827) and Cx3cr1CreER/+ (Stock No: 021160). The Pten+/- strain was from the National Cancer Institute. The Rosa26Eif4E strain was generated using a C57BL/6 embryonic stem cell line and maintained on the C57BL6 genetic background. Sex and age of animal were specified on methods for different assays.
Wild animals	This study did not involve wild animals.
Field-collected samples	This study did not involve samples collected from the field.
Ethics oversight	Animal care and experimental procedures were approved by Scripps Florida Institutional Animal Care and Use Committee (protocol # 16-003), according to US National Institutes of Health Guidelines (Online Methods/mouse husbandry).

Note that full information on the approval of the study protocol must also be provided in the manuscript.
