# Supplementary material for: Elevated protein synthesis in microglia causes autism-like synaptic and behavioral aberrations
Source: Nat Commun. 2020 Apr 14;11:1797. doi: 10.1038/s41467-020-15530-3 (PMC7156673; doi:10.1038/s41467-020-15530-3)
Supplement: Supplementary file 2 — Peer Review File [file 41467_2020_15530_MOESM2_ESM.pdf]

Editorial Note: This manuscript has been previously reviewed at another journal that is not operating a transparent peer review scheme. The manuscript was considered suitable for publication without further review at *Nature Communications*.
